# Cytotoxic T Cells: Kill, Memorize, and Mask to Maintain Immune Homeostasis

**DOI:** 10.3390/ijms26188788

**Published:** 2025-09-09

**Authors:** Vijay Kumar

**Affiliations:** Laboratory of Tumor Immunology and Immunotherapy, Department of Surgery, Morehouse School of Medicine, Atlanta, GA 30310, USA; vij_tox@yahoo.com or vijkumar@msm.edu; Tel.: +1-470-670-6942

**Keywords:** CD8^+^ cytotoxic T cells, immune homeostasis, Gzms, perforin, cytotoxic, infection, cancer

## Abstract

Homeostasis must be maintained for the healthy living of an organism. In addition to physiological and anatomical homeostasis, the maintenance of the immune system, called immune homeostasis or immunohomeostasis, is critical for overall well-being and general homeostasis. CD8^+^ cytotoxic T cells/lymphocytes (CTLs) are crucial components of the adaptive immune systems of all vertebrates with a thymus. Hence, the thymus is an essential primary lymphoid organ (PLO) for developing T cell-mediated immunity (TCMI) that comprises CD4^+^ helper T cells (Th) cells and their subtypes, such as Th0 (naïve helper T cells), Th1 (pro-inflammatory Th cells that secrete IFN-γ), Th2 (secrete type 2 cytokines, such as IL-4, IL-5, IL-6, IL-10, and IL-13), Th9 (secrete IL-9), Th17 (secrete IL-17), Th22 (secrete IL-22), follicular Th cells (T_fhs_, secrete IL-21), regulatory T cells (T_regs_), and CD8^+^CTLs. The current article explores the critical role of CD8^+^CTLs in the maintenance of immune homeostasis. The role of the thymus (PLO) in generating and regulating CD8^+^CTLs, as well as mobilizing them to distant lymph nodes (LNs) and the spleen, which are referred to as secondary lymphoid organs (SLOs) and target organs, is discussed in section two of the article. The subsequent third section discusses the role of CD8^+^CTLs’ cytotoxic and immunoregulatory action to maintain immune homeostasis during infection and other inflammatory conditions. Moreover, they mask themselves to different cell types, like Th cells, such as Tc2s, Tc9s, Tc17s, and Tc22s, to maintain immune homeostasis. CD8^+^CTLs also behave as T_regs_ to exert their immunoregulatory functions. In addition to conventional CD8^+^CTLs, granzyme K (GzmK)^+^CD8^+^CTLs and CD4^+^CTLs with their cytotoxic action to maintain immune homeostasis have also been discussed. The next section discusses cell–cell (APC–CD8^+^CTL) interactions that not only increase the cytotoxic functions of CD8^+^CTLs but also program APCs to support their cytotoxic functions. These CD8^+^CTLs secrete different cytokines (IFN-γ and IL-10) and cytotoxic molecules (perforin and Gzms), which exert immunoregulatory actions to maintain immune homeostasis. The article concludes with a future perspective and a conclusion section, highlighting the critical need to understand CD8^+^CTLs’ cytotoxic and immunoregulatory functions in maintaining immune homeostasis across various diseases, including those with newly identified roles for CD8^+^CTLs.

## 1. Introduction

Homeostasis is characterized as any self-limiting biological/physiological process maintaining the organism’s well-being by modulating physiological processes optimal for survival. Failure to maintain homeostasis leads to the development of a disease, which, if not treated, can cause severe organ damage and death as a result.

Immunological well-being that further controls an individual’s homeostasis to maintain his/her health status via maintaining the optimum immune function that fights external and endogenous threats is called **immune homeostasis** or **immunohomeostasis** [1]. For example, the local tissue immune microenvironment comprises residential immune cells, such as tissue-resident macrophages, dendritic cells (DCs), innate-lymphoid cells (ILCs), and different types of T cells, including tissue-resident memory T cells (TRMs), which are guardians of organ homeostasis [2,3,4]. However, the aged brain and the immune system disturb this organ homeostasis and decrease an individual’s longevity by promoting aging, inflammation, and mortality [5,6,7]. Hence, aging is another critical factor that disturbs the immune homeostasis to induce and support a chronic organ-specific local or systemic inflammation that may support cancer development, cardiometabolic diseases, chronic kidney disease (CKD), and neurodegeneration [8,9,10,11]. The dysregulated or derailed immune response is responsible for several pathologies, such as infectious diseases, allergies, cancers, neurodegenerative, autoimmune, autoinflammatory, immunodeficiency, and other inflammatory diseases, including metabolic disorders. Furthermore, teaching self-tolerance prevents self-attack by the immune system for maintaining homeostasis [12,13]. The events altering immune homeostasis may have a long-lasting impact through various mechanisms, including immune imprinting, reprogramming, reconfiguration, and remodeling, which can increase disease susceptibility [14].

For example, high mortality among patients with comorbidities (lifestyle-related diseases and cancer) and increased age (associated with dysregulated immune homeostasis) in the most recent Coronavirus disease-2019 (COVID-19) pandemic caused by severe acute respiratory syndrome coronavirus-2 (SARS-CoV-2) is an excellent example indicating the importance of maintaining immune homeostasis [15,16,17]. Hence, it is critical to understand immune homeostasis. The cytotoxic T cells/lymphocytes (CTLs) of the adaptive immune system are critical components of the vertebrate immune system. These cytotoxic T cells exist as naïve, effector, central memory, and tissue resident memory immune cells to fight against invading pathogens and antigens (Ags) and other inflammatory/immune-mediated diseases, such as cancers, autoimmunity, and neurodegenerative diseases (NDs). Therefore, the current article discusses the role of cytotoxic T cells in immune homeostasis.

## 2. Thymus, a Critical Site for T Cell Development

**Figure 1:**
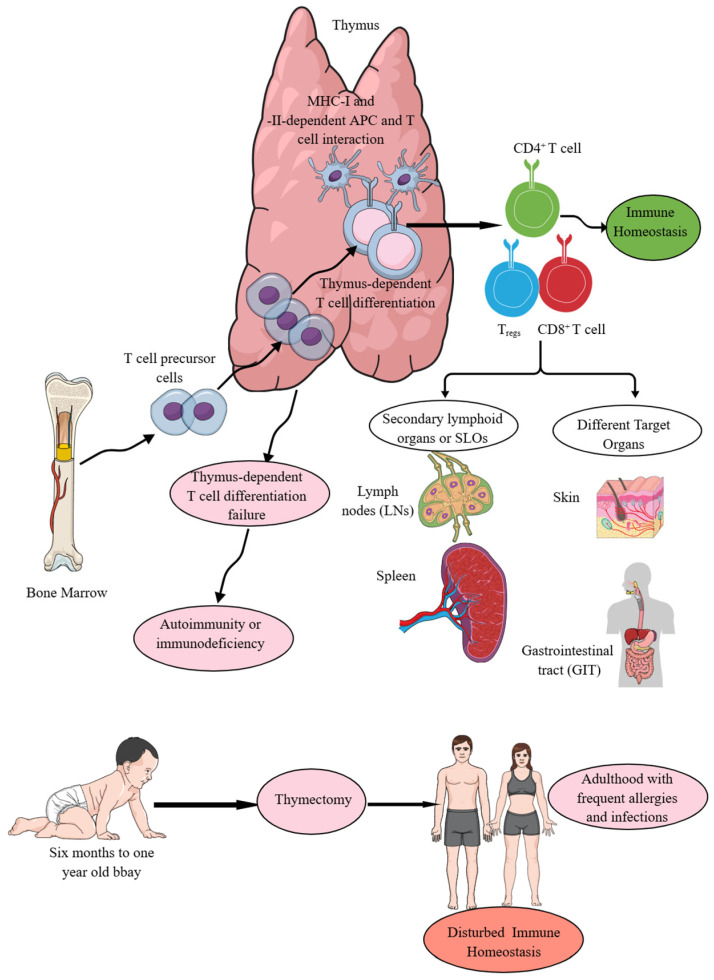
The thymus is central to immune homeostasis. T cell precursors move from the bone marrow (BM) to the thymus. In the thymus, thymus-dependent T cell differentiation processes give rise to T cells, which further, depending on MHC-I and MHC-II Ag presentation, develop into CD4^+^ helper T cells and CD8^+^ cytotoxic T cells. Moreover, T_reg_ production also occurs in the thymus. The failure of thymus-dependent T cell differentiation processes predisposes to autoimmunity or immunodeficiency disorders depending on the functional status of the T cells. From the thymus, T cells migrate to SLOs and other non-lymphoid target organs to perform their immune functions to maintain immune homeostasis. Details are mentioned in the text.

The thymus is one of the major thoracic organs in fetuses and neonates, as is evident when they are subjected to anatomical or histological examination. In adults, it undergoes fatty involution from puberty onwards [18]. The thymus serves as an immune organ, as indicated by its critical role in the development of T cell-mediated immunity (TCMI) in vertebrates (Figure 1), and histologically, it comprises thymic epithelial cell subsets, T cells, B cells, macrophages, DCs, and myoid cells [19,20]. The expression of antigen-specific diverse T cell receptors (TCRs) involving the recombination of germline-encoded gene segments, thymic education comprising negative selection of potentially autoreactive T cells, and positive selection of T cells capable of recognizing external Ag encountered at peripheral surfaces and circulation are called thymus-dependent T cell differentiation processes, which are critical for TCMI and immune homeostasis maintenance (Figure 1) [21,22,23,24,25,26,27,28]. Thymic cortical epithelial cells (cTECs) are critical APCs to promote positive selection among developing thymocytes in the thymus [29]. These cTECs produce self-peptides presented by major-histocompatibility complex-encoded molecules (pMHC) due to the expression of unique sets of proteases and, consequently, they express a unique set of pMHC complexes for Ag presentation to developing thymocytes for promoting positive selection [29,30,31].

MHC-II- and MHC-I-specific T cell receptor (TCR) signals generate CD4^+^ helper T (Th) and CD8^+^CTLs during the thymic selection process that depend on the T cell specificity and function (Figure 1) [32]. The thymus-dependent T cell differentiation process generating CD4^+^ and CD8^+^T cells (helper and cytotoxic T cells) depends on T cell lineage fate that is determined by *cis*-regulatory elements in coreceptor gene loci and is not determined by the coreceptor proteins they encode, invalidating coreceptor signal strength as the basis of lineage fate determination [33]. Hence, signal length but not the signal strength is a critical determinant of the T cell fate [33,34]. Moreover, the first wave of T cell development in the thymus generates CD4^+^T cells from all thymocytes via TCR signaling that is followed by a second TCR signaling wave, which coincides with CD8^+^T cell lineage specification [35]. Interestingly, in the thymus, the TCR–calcineurin–nuclear factor of activated T cell (NFAT)–GATA-binding protein-3 (GATA3) axis is a major driver of CD4^+^T cell fates [35].

The thymocytes bearing autoreactive TCRs either undergo clonal deletion or differentiate into specialized regulatory T (T_regs_) in response to IL-2, which is released from mature CD4 single-positive (CD4SP) thymocytes, along with the presence of IL-15 or CD4^+^ effector T cells (T_effs_) [36,37,38,39,40]. T_regs_ production in the thymus medulla is governed by transforming growth factor-β (TGF-β) expression that induces forkhead box P3 (Foxp3 or scurfin) expression, responsible for T_regs_ development by disrupting weaker agonist signals [41,42,43]. Moreover, this Foxp3 expression occurs independent of IL-2 except under non-physiological conditions in vivo [41]. The autoreactive T cells, which escape clonal deletion due to the absence of CD28 co-stimulation or transgenic overexpression of the antiapoptotic molecules, such as B cell lymphoma-2 (Bcl-2), Bcl-6 or myeloid cell leukemia-1 (Mcl-1), along with the surviving thymocytes [thymic PD1^+^ intraepithelial lymphocyte precursors (IELps)], differentiate into anergic CD4^−^CD8^−^ thymocytes positive for the TCRαβ subtype and then preferentially migrate to the intestine, where they re-express CD8α and sequester as CD8αα^+^ IELs [40,44,45]. Hence, CD28 co-stimulation signaling as an intrathymic signal is essential for clonal deletion to get rid of autoreactive T cells, and CD4^−^CD8^−^ TCRαβ subtypes of thymic T cells escaping this clonal deletion process move to the intestine and acquire the CD8αα^+^ IEL phenotype [46,47]. Interestingly, thymic *PDCD1*^+^ cells in the human thymus serve as putative CD8αα precursors and have a biased TCR repertoire with enriched large and positively charged complementarity-determining region 3 (CDR3) amino acids [48]. Moreover, CD28 signaling also promotes target organ-specific localization of T cells following their priming, as the absence of CD28-dependent signaling fails to localize primed T cells to non-lymphoid organs [49,50,51]. This CD28 signaling-dependent migration of primed/memory T cells from lymphoid organs to non-lymphoid antigenic sites occurs independent of TCR signaling and homing receptor expression [52]. Notably, CD28–CD152 or CTLA4 (cytotoxic T lymphocyte-associated antigen protein 4) interactions inhibit this migration of primed T cells from lymphoid organs to non-lymphoid organs or antigenic sites [52]. The leukemia/lymphoma-related factor (LRF) is critical for integrin β7 expression on thymic IELps and the gut homing of CD8αα^+^IELs, as mice thymic IELps lacking LRF do not generate TCRαβ^+^CD8αα^+^ IELs and their CD8β-expressing counterparts, despite giving rise to thymus and spleen CD8αβ^+^ T cells [53].

The CD4^−^CD8^−^ TCRαβ subtypes of thymic T cells exhibit a distinct TCR expression pattern that recognizes an unusual pattern of MHC restriction that is nonoverlapping with that of CD4^+^ or CD8αβ^+^ T cells; therefore, they recognize antigens escaping recognition by conventional T cells [54,55]. Hence, CD8αα^+^ IELs recognize different Ags, which are not recognized by conventional T cells due to their different TCRαβ, which lack CD4 and CD8αβ co-receptors’ expression [56]. Moreover, medullary thymic epithelial cells (mTECs), thymic DCs, and thymic B cells acquire functional adaptations to develop central tolerance for avoiding the development of autoreactive or autoimmune T cells as discussed in detail elsewhere [57,58]. For example, CD8^+^T cell tolerance to T cell-derived inflammation-associated self-Ags is efficiently induced in the thymus and supported by redundancy in cell types expressing these molecules [59]. TECs, thymic eosinophils, and innate-like T cells are critical for inducing CD8^+^T cell tolerance to T cell-derived inflammation-associated self-Ags and removing autoreactive CD8^+^ thymocytes/T cells in the thymus [59,60,61]. Further studies have indicated that the inflammatory thymic ‘ecosystem’ or thyminflammation has evolved to support and promote immune tolerance to ‘inflammatory self’-endogenous molecules absent from most peripheral tissues at steady state but upregulated during pathogen invasion [62]. Moreover, thymic mimetic cells (TMCs) have the potential to differentiate into different types of molecularly distinct and functionally defined cells, such as well-defined parenchymal cells, including endocrine cells, microfold cells, and myocytes, which not only contribute to the induction of central tolerance but also regulate the homeostasis of other thymus-resident populations [63].

The thymus is the primary site of T cell development and maturation (Figure 1), although extrathymic (liver, intestinal cryptopatches, and lymph nodes) T cell development has also been reported [22,64,65,66,67,68,69]. However, a later study comprising fate mapping of retinoic acid receptor (RAR)-related orphan receptor-γt (RORγt)^+^ cells has indicated that all intestinal αβT cells are the progenies of CD4^+^CD8^+^ thymocytes and supports that the adult intestine or intestinal cryptopatches, which are lymphoid aggregates induced by lymphoid tissue-inducing (LTi) cells and are required for intestinal immune responses, are not the genuine sites of ab T cell development [66]. These extrathymic T cells share critical features, such as activated phenotype, precocious expansion, and production of copious amounts of IFN-γ with MHC-Ib-restricted T cells (Qa-1-, Qa-2-, and M3-restricted T cells) to fight against invading pathogens [70,71,72,73,74]. The exclusion of extrathymic development of αβ^−^T cells in intestinal cryptopatches further supports the critical role of the thymus in T cell development and maturation, providing both systemic and local (organ-specific) TCMI in all jawed animals, which are all vertebrates with adaptive immunity [75]. Moreover, the thymus has been conserved for over 450 million years of evolution, and no equivalent or substitute is available in the animal kingdom, unlike the presence of ten different organs for hematopoiesis in gnathostomes [75].

The thymus-dependent T cell differentiation processes (CD4^+^T helper cells, CD8^+^ killer or cytotoxic T cells, and CD4^+^T_regs_) ensure the recognition of foreign Ags in context with the recognition of self-MHC or human leukocyte antigen (HLA) molecules (MHC-I or HLA-1 and MHC-II or HLA-II) expressed on different antigen-presenting cells (APCs, such as DCs, macrophages, and B cells) without inducing self-reactive autoimmunity along with supporting the development of several minor T cell subsets that promote immune and tissue homeostasis (Figure 1) [76]. Failed thymus-dependent T cell differentiation processes lead to T cell-dependent immunodeficiencies or autoimmune diseases (AIDs) due to a breach in immune homeostasis (Figure 1) [22,77]. Moreover, children undergoing thymectomy during the first six months of their life suffer from T cell lymphopenia (premature immune aging) that predominantly affects their naïve T cells as the thymus is the primary site for their development and they exhibit decreased T cell (CD4^+^ helper and CD8^+^ cytotoxic T cells) diversity 18 years after thymectomy and suffer more episodes of infections and allergies (Figure 1) [78,79]. Hence, the thymus is a critical primary lymphoid organ (PLO) that provides TCMI to jawed vertebrates, and its absence or underdevelopment at the time of birth and removal at an early age (<1 year) disturbs immune homeostasis and predisposes the host to different diseases, such as frequent episodes of infections and allergies (Figure 1). The details of different types of CD4^+^T cells, such as helper T (Th) cells and their subtypes, which develop in the thymus and move to specific organs for their particular organ-specific functions, are beyond the topic of the current article and have been discussed in detail elsewhere [80,81,82,83,84,85]. The focus of the current article is cytotoxic T cells and their contribution to maintaining immune homeostasis.

## 3. Cytotoxic T Cells or CTLs in Adaptive Immunity Regulating Infection and Inflammatory Processes

### 3.1. Conventional CD8^+^ Cytotoxic T Lymphocytes/Cells (CD8^+^CTLs)

**Figure 2:**
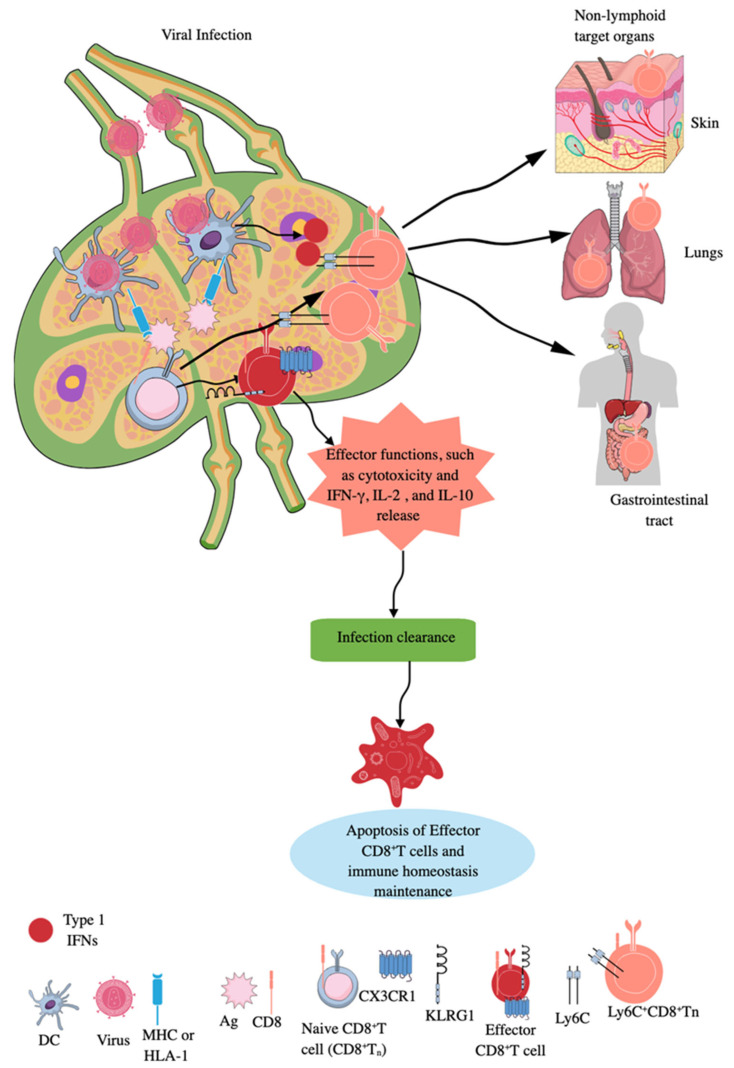
CD8^+^CTLs during infection. Naïve CD8^+^CTLs are primed by APCs expressing MHC-I molecules, which uptake the virus and present the processed Ag to them. The effector CD8^+^CTLs expressing KLRG1 and CX3CR1 exert their cytotoxic action to clear the infection. Infection clearance induces apoptosis of effector CD8^+^CTLs to maintain immune homeostasis and naïve CD8^+^CTLs are expanded to unique long-lived Ly6c^+^CD8^+^T_n_ cells, which exert potent cytotoxic action in SLOs and move to non-lymphoid organs or antigenic sites in response to type 1 IFNs to take care of future infections. Kindly see the text for details.

CD8^+^T cells are classic adaptive immune killer or cytotoxic T cells, which target intracellular infections and transformed cancer cells, and also play a critical role in AIDs [86]. During an infection, the priming of naïve CD8^+^T cells by APCs, such as DCs, macrophages, and B cells, occurs in secondary lymphoid organs (SLOs), like lymph nodes (LNs) and spleen (Figure 2) [87,88]. LNs are critical for immune surveillance and continuously receive cells, molecules, antigens, and pathogens from tissues via afferent lymphatic vessels to guide future adaptive immune responses to clear these pathogens before their systemic distribution (Figure 2) [89,90]. Naïve CD8^+^T cells interact with Ag-laden MHC-1 molecules of DCs in the subcapsular sinus region or interfollicular region of the draining lymph node (DLN) to become effector CD8^+^T cells (highly killer cell lectin-like receptor G1 (KLRG1) and CX3CR1 positive), exerting their cytotoxic action (Figure 2) [89,91,92,93,94].

A different subset of CD8^+^CTLs has been identified in patients with leprosy (caused by *Mycobacterium leprae*), expressing natural killer (NK) cell receptors NKG2C (NKG2-C type II integral membrane protein) and NK Group 2 family of receptor A (NKG2A or CD159a, which form heterodimers with CD94 and trigger stimulatory action upon activation signaling) along with granzymes (Gzms), granulysin (not expressed in mice), and perforin and kill macrophages infected with *M. leprae* [95]. Nevertheless, activation of only NKG2C on these CD8^+^CTLs induces the release of Gzm, granulysin, and perforin to exert cytotoxic action on macrophages infected with *M. leprae* in a TCR-dependent and independent manner. Notably, NKG2A activation blocks the cytotoxic action of these CD8^+^CTLs. Moreover, these NKG2C^+^CD8^+^CTLs have greater antimicrobial action against intracellular bacteria, such as *M. leprae* and *Listeria monocytogenes* (*L. monocytogenes*), than conventional effector CD8^+^T cells [95]. Interestingly, Langerhans cells (LCs), but not CD14^+^DCs in the skin, are more potent to differentiate naïve e CD8^+^T cells (T_n_) into CD8^+^CTLs [95].

During infection, T_ns_ can also expand into unique long-lived Ly6c^+^CD8^+^Tn cells, which have accelerated effector function in SLOs, such as LNs (Figure 2) [96]. Type 1 IFNs promote this expansion of long-lived Ly6c^+^CD8^+^Tn cells with robust effector functions during infections, as they upregulate MHC-I expression and enhance tonic TCR signaling in differentiating T_n_ cells [96]. Furthermore, type 1 IFN-mediated signals optimize Ly6c^+^CD8^+^T_n_ cells’ homing to secondary sites (non-lymphoid organs or antigenic sites), extend their lifespan, and increase their effector differentiation and antimicrobial functions (Figure 2). However, the majority of effector CD8^+^T cells die via apoptosis, which is promoted by transforming growth factor-β (TGF-β), once the infection is cleared (Figure 2). In contrast, a low number of these cells differentiate into memory precursor (MP) cells expressing IL-7R, where IL-7 and IL-15 promote their survival and proliferation [97,98,99,100]. The details about tissue-resident CD8^+^ memory T cells and their roles in human pathologies, such as infections and cancers, have been discussed elsewhere [101,102,103,104]. Interestingly, memory CD8^+^T cells (central and tissue-resident CD8^+^ memory T cells) of sepsis survivors exhibit altered transcriptional profiles and chromatin accessibility, indicating long-lasting T cell intrinsic changes that decrease their potential to fight against bacterial (*Listeria monocytogenes*) and viral infections and cancer [105,106,107,108]. However, sepsis severity critically determines the degree of impairment among memory CD8^+^T cells of sepsis survivors [106].

The canonical/classic CD8^+^ cytotoxic T cell activation also involves different co-stimulatory molecules (CD28 receptor expressed on CD8^+^T cells interacts with CD80/B7.1 or CD86/B7.2 of APCs, which lowers the stimulation threshold of naive CD8^+^T cells and enhances cell proliferation and cytokine production) and the CD8 co-receptor along with the recognition of Ag molecules bound to the MHC-I molecules expressed on different APCs and the activation of their TCRs, forming an immune synapse between APC and CD8^+^T cell by utilizing their supramolecular activation complex and adhesion molecules (such as intercellular adhesion molecule or ICAM) on the target cell surface [109,110]. Moreover, to achieve full expansion along with this immune synapse between APC and CD8^+^T cells, inflammatory cytokines, like IL-12 and type 1 IFNs, are also critical [111,112].

The CD28 involvement activates downstream phosphatidylinositol 3 kinase (PI3K) and mammalian target of rapamycin (mTOR)-mediated activation of protein kinase B (PKB/Akt) and nuclear factor-κB (NF-κB), culminating in the overexpression of an antiapoptotic molecule called B cell lymphoma extra-large (Bcl-xL) and increasing cytotoxic T-cell survival [113,114,115,116,117]. This event prepares CD8^+^T cells for their cytotoxic action by enhancing pore formation in the target cell membrane and secreting killing granules, which are secretory lysosomes loaded with Gzms, perforin, cathepsin C, and granulysin, that fuse with the target cell membrane to induce death [118,119,120,121]. Perforin exocytosis and target cell permeabilization occur in as little as 30 s, allowing for the rapid diffusion of extracellular milieu-derived Gzms into target cells, where they deliver their lethal amount and induce cell death [122]. It is interesting to note that repair of these pores in cytotoxic T cells starts within 20 s and completes in 80 s to limit Gzm diffusion [122]. The pores formed by perforin resemble the complement-induced membrane attack complex (MAC, a C5b, C6, C7, C8, and C9 complex). However, perforin pores have perforin domains and are heterogeneous and larger in diameter (~16–21 nm) than MAC (~10 nm) [123,124,125,126,127]. These perforin-induced pores induce uncontrolled influx of extracellular small molecules, especially cations, such as Ca^2+^, and induce osmotic stress to cause colloid osmolytic cell death of the target cell, along with serving as a conduit for CTL-cytotoxic proteins, such as Gzms [123,124,126,127,128].

Moreover, perforin induces rapid plasma membrane phospholipid (phosphatidylserine) flip-flop that may provide a flexible gateway for GzmB to translocate across the bilayer to the cytosolic leaflet of target cells to induce the cell death [129]. The target cell’s membrane with extracellular phosphatidylserine is the point where perforin pores are formed to inject Gzms [130]. Therefore, performin forms arc-like structures on target cell membranes, which serve as minimally disrupting conduits for GzmB translocation [130]. Moreover, along with membrane flip-flop and arc formation in the target cell membrane, perforin also induces invagination and vesicle formation prior to pore formation for delivering Gzms [131,132]. Gzms, such as GzmB, cleave their substrate proteins, including caspase 3 (CASP3 or CPP32) and CASP7, after their aspartic acid residues to initiate target cells’ apoptosis [133,134]. The apoptosis of the target cell starts within 2 min of perforin permeabilization [122].

GzmA also induces CASP-independent cell death by cleaving mitochondrial complex 1 protein (NDUFS3, an iron-sulfur subunit of the NADH: ubiquinone oxidoreductase complex I) to produce reactive oxygen species (ROS) that disrupt transmembrane potential [135]. The details about Gzms, cytotoxic mechanisms of action, and their role in immunity and inflammation have been discussed elsewhere [136,137,138,139,140,141]. In addition to their direct cytotoxic actions, endocytosis of CD8^+^ cytotoxic T cell membranes by target cells also loads target cells with killing enzymes and proteins, such as Gzms, perforin, and granulysin, to induce their death [136,142,143].

The pores formed by perforin and granulysin in the endosomal membranes subsequently release cytotoxic Gzms in the cytosol. CD8^+^CTLs also use their Fas ligand (FasL or CD95L) to interact with the Fas (CD95) receptors of target cells to induce their apoptotic cell death by activating the Fas-associated death domain (FADD), which activates downstream caspases, critical for apoptosis [120,144]. However, the target cell determines its fate during CTL-mediated cytotoxicity by triggering the repair response to restore membrane integrity to avoid necrosis and undergo the slow process of apoptotic cell death [145]. For example, perforin activates clathrin- and dynamin-dependent endocytosis, which removes perforin and Gzms from the plasma membrane to early endosomes, preserving outer membrane integrity. Inhibiting clathrin- or dynamin-dependent endocytosis shifts death by perforin and GzmB from apoptosis to necrosis [146]. Perforin disruption of the plasma membrane induces a transient Ca^2+^ flux into the target cell that triggers a wounded membrane repair response in which lysosomes and endosomes donate their membranes to reseal the damaged membrane [145].

Perforin-deficient mice exhibit impaired clearance of viruses, such as lymphocytic choriomeningitis virus (LCMV), due to the defective function of CD8^+^CTLs and natural killer (NK) cells, despite maintaining their normal numbers [147]. It is interesting to note that the N-linked glycosylation of the perforin C-terminus at Asn549 within the endoplasmic reticulum (ER) inhibits oligomerization of perforin monomers, which protects the host cell from premature pore formation and self-destruction [148]. The deglycation of the perforin occurs only inside the secretory granule/lysosome via its proteolytic processing of the C-terminus, which is imperative for perforin activation prior to secretion [148]. Thus, post-translational regulatory mechanisms are critical to maintain the inactive form of the perforin until its secretion from the inhibitory acidic environment of the secretory granule/lysosome is required [148]. The details of perforin structure, cytotoxic functions, and role in immunopathogenesis of human diseases, including cancers, are discussed in detail elsewhere [149,150,151,152].

On the other hand, the reciprocal TCR–MHC–peptide complex recognition polarity impedes TCR signaling and thus, CD8^+^T cell activation occurs due to the mislocalization of CD3 and CD8 (co-receptor)-associated lymphocyte-specific protein tyrosine kinase (Lck) [153]. The MHC-peptide complex and TCR signaling have been described in detail elsewhere [154,155]. Ag exposure to CD8^+^T killer/cytotoxic cells induces their robust expansion to generate effector and memory T cells [80]. CD29 expression on CD8^+^CTLs is a critical marker for their cytotoxic potential, as it is highly expressed on highly cytotoxic CD8^+^CTLs producing IFN-γ isolated from patients with melanoma [156,157]. Moreover, CD8^+^CTLs, due to their potent anticancer action, form the backbone of currently available cancer immunotherapies, such as immune-checkpoint inhibitors (ICIs) and chimeric-antigen receptor (CAR) T cell therapies [109,158,159].

Chronic/persistent viral infections (CVIs) and cancer development, progression, and metastasis are supported by the suppression of the antiviral and anticancer functions of CD8^+^CTLs. For example, CD160^+^CD8^+^ effector T cells increase in number in patients with chronic lymphocytic leukemia (CLL), which represent the phenotype of exhausted CD8^+^T cells [160]. These patients with CLL have increased circulating IL-6 levels, which upregulate CD160 expression among effector CD8^+^T cells and their exhaustion. Along with CD160, these CD8^+^T cells also overexpress other exhaustion markers (CD244 and programmed cell death protein 1 or PD1) and lose their cytotoxic potential due to defective Gzm packaging in vesicles and proliferation capacity, but retain their ability to secrete cytokines, such as IFN-γ, TNF-α, and IL-2 [161]. The details about exhausted CD8^+^CTLs in cancers and CVIs have been discussed elsewhere [162,163,164,165,166,167].

Interestingly, the expression of Kruppel-like factor 2 (KLF2) TF prevents the generation of exhausted CD8^+^CTLs, maintaining their lineage fidelity and polyfunctional tumor-specific progenitor traits by suppressing thymocyte selection-related HMG (high mobility group) box protein (TOX) TF, responsible for exhausted CD8^+^CTL generation, and upregulating T-bet TF expression [168]. Additionally, exhausted CD8^+^CTLs downregulate IL-18 receptor (IL-18R) expression and do not respond to pro-inflammatory cytokines, such as IL-12, IL-18, and IL-21, to exert their antiviral and antitumor function [169]. Thus, strategies increasing the expression of KLF2 and IL-18R in exhausted CD8^+^CTLs may help to reprogram them to effector CD8^+^CTLs to fight against CVIs and cancers.

Even so, developing a vaccine promoting CD8^+^T cell memory against viral infections and cancers is a difficult task due to their heterogeneity, which arises due to their progressive loss of effector functions, overexpression of immune checkpoints/inhibitory receptors, dysregulated transcriptional and epigenetic programming mechanisms, and unknown signaling mechanisms responsible for their development [170]. Interestingly, terminally exhausted CD8^+^T cells expressing T cell factor 1 (TCF-1) TF exhibit stem cell-like characteristics, similar to memory T cells, and their development indicates the adaptability of CD8^+^T cells to the continuous availability of Ag during CVIs and cancers [170,171,172,173]. Moreover, recent vaccine strategies that transiently inhibit type 1 IFN by blocking the IFN-α receptor (IFNAR) have been shown to increase the stemness of CD8^+^T cells, as indicated by the differentiation of TCF-1^+^ stem cell-like memory CD8^+^ T (TSCM) cells [171,173,174]. This strategy has increased the efficacy of vaccines and ICIs against CVIs and cancers, such as melanoma, due to increased Ag load, a counterintuitive increase in IFN-γ, and proliferation of TSCMs.

For example, CD101^−^Tim3^+^ CD8^+^T cells generated from TSCMs upon PD-1-based ICIs during CVIs highly express CX3CR1 and GzmB, but become TCF-1^−^ and T-bet^+^ and exhibit their antiviral actions [175]. ICI-mediated CX3CR1 expression is governed by histone deacetylase 1 (HDAC1) in TSCMs to generate effector CD8^+^CTLs, such as CD101^−^Tim3^+^CD8^+^T cells, to fight against CVIs [176]. Moreover, the PD-1-specific ICIs in combination with IL-2 have exerted greater beneficial effects in CVIs, such as chronic LCMV infection by generating highly active effector CD8^+^CTLs, which overexpress the high-affinity IL-2 trimeric (CD25-CD122-CD132) receptor, which does not occur during PD-1-specific ICI only treatment [177,178,179]. The interaction between CD25–IL-2 is critical for the beneficial synergistic effect of the PD-1-specific ICI and IL-2 combination, which has shown considerable activity in cancer patients [177,180,181]. However, to avoid the unwanted systemic effects from IL-2 therapy due to the constitutive expression of CD25 on Tregs and endothelial cells, PD-1 cis-targeted IL-2R agonists have been developed [182]. The details of TSCMs, which are rare memory T cells with the ability to reconstitute the entire spectrum of memory and effector T cell subsets with therapeutic potential, have been discussed in detail elsewhere [183,184,185,186].

The virus-specific stem-like CD8^+^T cells develop within five days post-CVI caused by LCMV, irrespective of the infection outcome [187]. Virus-specific stem-like CD8^+^T cells also develop during acute LCMV infection, like those in chronic LCMV infection. However, transfer of stem-like CD8^+^T cells generated five days post CVI to mice with acute viral infection (AVI) induces the downregulation of canonical markers of the chronic stem-like CD8^+^T cells and upregulates the expression of central memory T cell markers (CD127 and CD62L) [187]. On the other hand, when virus-specific stem-like CD8^+^T cells generated five days post AVI are transferred to mice infected with LCMV-induced CVI, they behave like virus-specific stem-like CD8^+^T cells generated during CVI and respond well to PD-1 inhibitors (ICIs), indicating the need for prior preparation of the host to deal with CVIs. Interestingly, other prior studies have indicated that progenitor exhausted CD8^+^CTLs (PD-1^intermediate^CXCR5^+^TCF-1^+^), like TSCMs, are more responsive to ICIs than terminally exhausted CD8^+^CTLs [188,189].

The progenitor exhausted CD8^+^T cells (T_pex_) overexpress inducible co-stimulator (ICOS) molecules, which interact with ICOS ligand (ICOSL) on APCs to suppress forkhead box O1 (FoxO1) TF expression, which suppresses the induction of memory-like features among T_pex_ cells but stimulates their transformation to terminally exhausted T cells (T_exs_) [190]. On the other hand, blocking the inducible costimulator (ICOS or CD278)–ICOS-ligand (ICOSL) interaction supports the generation of effector-like PD-1^+^CD8^+^T cells, reduces the viral load, and improves the response to PD-1 blockade in CVIs and cancer TME due to overexpression and overactivity of FoxO1, as it serves as a master regulator of memory programming in human CAR T cells [190,191]. In addition, FoxO1 expression also promotes a stem-like phenotype in CAR T cells that correlates well with their mitochondrial fitness and their persistence and therapeutic efficacy [192]. Thus, costimulatory molecules on T_pex_ cells also determine their fate toward generating memory-like features to take care of CVIs or inducing their exhaustion to promote CVIs and cancers.

During autoimmunity, CD8^+^ cytotoxic T cells become overactive due to their escape from different tolerogenic mechanisms and start to attack host cells to induce self-tissue damage, as seen during autoimmune and autoinflammatory disorders/diseases (AIDs) [86,193,194,195]. Type 1 IFNs increase the cytotoxic function of T cells by upregulating IFN regulatory factor 7 (IRF7) expression, which is responsible for GzmB production in AID, such as autoimmune kidney disease in mice and systemic lupus erythematosus (SLE) in humans and associated glomerulonephritis [196]. The details of CD8^+^CTLs in different AIDs have been discussed elsewhere [193,194,195].

### 3.2. Polarized/Masked CD8^+^CTLs with Lower/Lost Cytotoxic Potential but Secreting Different Pro- and Anti-Inflammatory Cytokines

**Figure 3:**
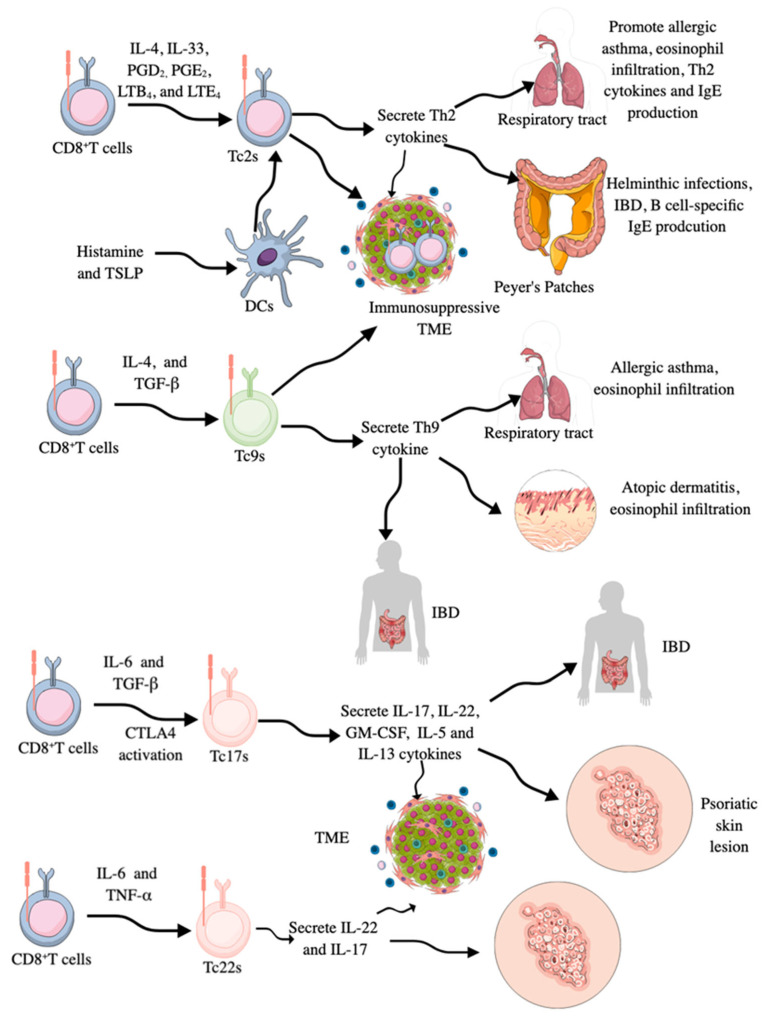
CD8^+^T cell masking as different types of helper T cells. CD8^+^T cells start to behave as Th2 cells called Tc2s by secreting Th2 cytokines in the presence of IL-4, IL-33, PGD_2_ PGE_2_ LTB_4_, and LTE_4_. Their number is increased in patients with asthma, patients with helminthic infections, and immunosuppressive TME/TIME. They increase B cell-specific IgE production, eosinophilia, and other Th2-based immune responses. Tc2s lose their cytotoxic activity. Tc9s are IL-9 secreting CD8^+^T cells generated in response to IL-4 and TGF-β. They also lose their cytotoxic activity. Their number is increased in patients with allergic asthma, atopic dermatitis, and IBD. Tc17s secrete IL-17, like Th17 cells. They also secrete IL-22, GM-CSF, IL-5, and IL-13. Their increased numbers have been reported in patients with IBD, psoriasis skin lesions, and different cancers. Tc22s secrete IL-22 and IL-17 and are generated from CD8^+^T cells in response to IL-6 and TNF-α. They are highly prevalent in the TME of patients with cancer and skin lesions of patients with psoriasis.

In addition to cytotoxic phenotype and function, CD8^+^CTLs may also behave as helper T cells and do not exhibit their cytotoxic activity. For example, CD8^+^CTLs secreting Th2 cytokines, such as IL-4, 5, 6, 10, and 13, have been detected in respiratory tract and interepithelial surfaces/tissues, such as Peyer’s patches, where they promote eosinophil infiltration, B cell-specific immunoglobulin E (IgE) production, and engage in allergic immune responses and are called **Tc2s** (Figure 3) [197,198]. The acute asthma exacerbation during virus-induced pulmonary airway infection occurs due to the polarization of conventional CD8^+^CTLs to Tc2s, which have lost their cytotoxic action against the virus (Figure 3) [198,199]. For example, IL-4-treated naïve CD8^+^CTLs produce IL-5 and IL-6 cytokines, and their IFN-γ production capacity is decreased (Figure 3) [200,201]. Moreover, in an experimental allergic inflammation model, the release of IL-33 promotes Th2 cytokine production from pulmonary CD8^+^CTLs, and these Tc2s are higher in the circulation of patients with severe asthma (Figure 3), which is associated with increased disease burden, higher exacerbation rates, and steroid insensitivity [202]. The treatment of Tc2s with prostaglandin D2 (PGD_2_), leukotriene B4 (LTB_4_), and LTE_4_, which are lipid mediators released by immune cells during allergic inflammation, also induces Th2 cytokines and chemokines, contributing to eosinophilia without the involvement of TCRs (Figure 3) [203,204,205,206].

PGE_2_ also suppresses the cytotoxic function of CD8^+^CTLs by inducing the expression of CD94/NKG2A (this heterodimer suppresses cytotoxic action upon interaction with MHC-1 gene products) via activating the cyclic adenosine monophosphate (cAMP)/protein kinase A (PKA) axis [207]. Moreover, histamine-treated DCs increase Tc2 infiltration in the lungs of allergic mice, where they produce Th2 cytokines to exaggerate the associated inflammatory immune response, such as increased pulmonary eosinophilia and increased serum IgE levels (Figure 3) [208]. Histamine also supports cross-presentation of soluble allergens by DCs, which further activates CD8^+^CTLs to aggravate the allergic immune response [209]. In addition to histamine, thymic stromal lymphopoietin (TSLP) stimulation of CD11c^+^DCs also activates and propagates Tc2s with no cytolytic/cytotoxic action (Figure 3) [210].

Even CD40L-triggering TSLP-stimulated DCs induces cytolytic CD8^+^T cells with cytotoxic action, but they retain their capacity to produce Th2 cytokines. Thus, TSLP serves as a primary signal for allergic T cell responses, and CD40L-expressing cells, such as DCs, may synergize with TSLP to amplify and sustain pro-allergic Th2 responses, causing tissue damage by promoting the generation of IFN-γ-producing cytotoxic effectors [210]. Hence, reprogrammed naïve CD8^+^CTLs producing Th2 cytokines called Tc2s are critical players in Th2 cytokine-associated inflammatory diseases, such as asthma, allergic rhinitis, allergic dermatitis, helminthic infections, and chronic intestinal inflammatory diseases, such as inflammatory bowel disease (IBD) (Figure 3) [211,212,213,214,215]. These CD8^+^CTLs with Th2 function are also reported in the immunosuppressive TME of different cancers, such as cervical and urothelial bladder cancers, due to the loss of cytotoxic action and acquisition of Th2 characteristics to support immunosuppressive TME due to the loss of their antitumor cytotoxic action (Figure 3) [211,216,217,218,219]. The differentiation of CD8^+^CTLs to Tc2s in the TME involves IL-4-mediated orthogonal IL-4 receptor (o4R) signaling activating the downstream Janus-associated kinase (JAK)–signal transducer and activator of transcription (STAT) signaling pathway [220]. Interestingly, oR4 expressing CD8^+^T cells are less prevalent in the TME but they are abundant in peripheral organs, influencing their expansion and differentiation into Tc2s and tumor infiltration [220]. Thus, the presence of Tc2s aggravates the inflammatory process associated with Th2-based inflammatory immune diseases but suppresses antitumor immunity required to clear cancer cells (Figure 3).

Moreover, CD8^+^CTLs may also secrete IL-9 and behave as IL-9-secreting Th9 cells called **Tc9s** (Figure 3). These Tc9s are transcriptionally regulated by STAT6 and IRF-4, and like CD8^+^CTLs secreting Th2 cytokines and Th2 cells, they develop in the presence of IL-4 and TGF-β and lose their cytotoxic action [221,222,223]. They are also critical drivers of allergy-associated pathologies, like allergic asthma and atopic dermatitis and associated eosinophilia and are seen in the TME of different cancers (Figure 3), such as breast cancer, where their anticancer action is governed by liver X receptor (LXR) activation in response to oxidized cholesterol, which inhibits IL-9 expression, which in turn inhibits Tc9 differentiation and function [222,224,225,226]. The Tc9-specific antitumor action of IL-9 involves self-activation of IL-9 receptors, which, via STAT3 activation, promote lipid peroxidation (LPO) or fatty acid oxidation (FAO) for their energy requirements and escape cancer cell-induced ferroptosis to exert antitumor action [227]. Therefore, Tc9s with less cholesterol have better anticancer action than those laden with high cholesterol [228].

IL-9 exerts different immunological actions depending on the site of action and the disease of its release. For example, during allergic asthma, IL-9 promotes asthma aggravating immune cell survival, recruitment, and proliferation, such as type 2 innate lymphoid cells (ILC2s), Th2 cells, B cells producing IgE, mast cells, and eosinophils, along with increasing mucus production, collagen deposition, and smooth muscle cell hyperplasia [229,230]. During IBD, IL-9 promotes the expression and function of pro-inflammatory cytokines, such as TNF-α, IL-5, and IL-13 release, along with increasing intestinal barrier permeability and suppressing tissue repair mechanisms [229]. In melanoma, TME IL-9 promotes mast cell and DC survival and IFN-γ production to increase the antitumor immune response [229,230]. For example, the adoptive transfer of tumor-specific Tc9s achieves long-term control of tumor growth by activating host CD4^+^T cells, which is also true in Ag-loss relapsed tumors [231,232]. The activation of antitumor CD4^+^T cells upon adoptive transfer of tumor-specific Tc9s occurs as a result of IL-24 secretion and recruitment of CCR7^+^cDC2s (conventional type 2 DCs) into tumor-draining LNs, which prime host CD4^+^T cells against relapsing tumors [232]. Moreover, the intratumoral IL-24 level correlates with cDC2 and CD4^+^T cell gene signatures in human cancers and better patient survival.

**Tc17s** are CD8^+^CTLs that secrete pro-inflammatory IL-17 cytokines along with IL-22, granulocyte-monocyte colony stimulating factor (GM-CSF), IL-5, and IL-13 (Figure 3), and are characterized by the expression of STAT3 and RORγt TFs [221,233,234,235]. IL-6 and TGF-β are critical factors for transforming CD8^+^CTLs into Tc17s (Figure 3). Moreover, CTLA4 stimulation in CD8^+^CTLs also promotes Tc17 generation [211]. IRF3 is a critical TF for IL-17 expression in Tc17 cells via its direct interaction with RORγt in the cytosol, which inhibits IL-17 expression in a type 1 IFN-dependent manner [236]. Tc17s have limited cytotoxicity due to very low GzmB and perforin levels [237]. However, treatment of Tc17s with IL-12 induces IFN-γ production by these cells along with IL-17 production, confirming them as IL-17/IFN-γ double-producing CD8^+^T cells (Tc17/IFN-γ) [238]. These IL-17/IFN-γ double-producing CD8^+^T cells (Tc17/IFN-γ) also acquire cytotoxic action and exhibit potent antitumor activity in vitro and in vivo, similar to effector CD8^+^CTLs. Thus, the tissue milieu, depending on the availability of cytokine type, potentially impacts the immunological functions of CD8^+^T cells and their cellular derivatives.

The presence of commensal microbiota-specific Tc17 cells indicates their critical role in tissue homeostasis, such as skin, lungs, and gut [233,234,239,240,241]. Moreover, Tc17 directly (via IL-17) and by antimicrobial peptide (AMP) production controls infections and inflammatory processes [211,242,243,244]. However, Tc17s are also increased in HIV-1-infected patients but lack antiviral action [245]. The pathogenic Tc17s increase in psoriatic skin lesions due to altered skin microbiota and differ from innate CD8^+^ mucosa-associated invariant T (MAIT) cells [235,246]. The details of Tc17 cells in inflammation and immunity in the context of infections, inflammatory diseases, autoimmunity, and cancers have been discussed elsewhere [233,247,248,249]. Interestingly, effector Tc17 cells do not undergo glycolysis, instead utilizing oxidative phosphorylation (OXPHOS) for their expansion upon fungal antigen challenge [250]. Hence, it will be interesting to investigate their immunometabolic reprogramming during other microbial infections, such as bacterial and viral infections, along with other inflammatory conditions.

CD8^+^CTLs secreting IL-22 are termed **Tc22s** (Figure 3), which, like Th22 cells, express aryl hydrocarbon receptor (Ahr) TF along with STAT1, 3, and 5 [211,221]. They develop in response to IL-6 and TNF-α-dependent Ahr activation in the TME and exhibit enhanced antitumor activity compared to conventional CD8^+^ CTLs, primarily due to increased cytotoxicity resulting from elevated GzmB activity [221,251]. In inflammatory conditions, such as atopic dermatitis and psoriatic skin lesions, Tc22 numbers increase and contribute to the disease pathology due to their pro-inflammatory actions, such as overproduction of IL-22, IL-17, and IFN-γ (Figure 3) [235,252,253,254]. However, in young children with pediatric atopic dermatitis (pAD), Th2 activation and their homing into the skin compartment are critical for disease pathogenesis and severity, but in adults, psoriasis, Th22, and Tc22s are critical for disease severity [255,256].

Tc22s also increase in people exposed to HIV-1 infection but do not develop symptoms due to a controlled viral titer that might be due to the increased antiviral/cytotoxic action of Tc22s relative to conventional CD8^+^CTLs, such as increased IL-22, IFN-γ, and GzmB release [257,258]. For example, IL-22 increases innate immunity against HIV infection via inducing the production of acute phase proteins, like acute phase serum amyloid A (A-SAA), which downregulates chemokine receptor 5 (CCR5, a HIV-1 entry receptor) expression by inducing its phosphorylation on APCs, such as DCs, to avoid their infection with the virus [259,260]. Thus, Tc22, due to its increased cytotoxic action and release of pro-inflammatory cytokines, such as IL-22 and IL-17, regulates TME/TIME and inflammatory processes during viral infections and other inflammatory diseases, such as atopic dermatitis and psoriasis. The antitumor action of Tc22s can also be attributed to their distinct metabolic demand compared to effector CD8^+^CTLs. For example, effector CD8^+^CTLs depend on glycolysis for their antitumor (IFN-γ release and cytotoxic action) action, whereas Tc22s rely on OXPHOS for differentiation and effector function as indicated by increased activity of the pantothenate/coenzyme A (CoA) pathway [261,262,263,264]. Moreover, exogenous treatment with CoA to CD8^+^T cells reprograms them to Tc22s by increasing their OXPHOS, which upregulates hypoxia-inducible factor-1a (HIF-1a) and Ahr activity and increases the antitumor action of Tc22s (IL-22 and IL-2 release) in murine cancer models and patients with melanoma receiving anti-PD-L1 ICIs [264].

### 3.3. CD8^+^ Regulatory T Cells (CD8^+^T_regs_)

**Figure 4:**
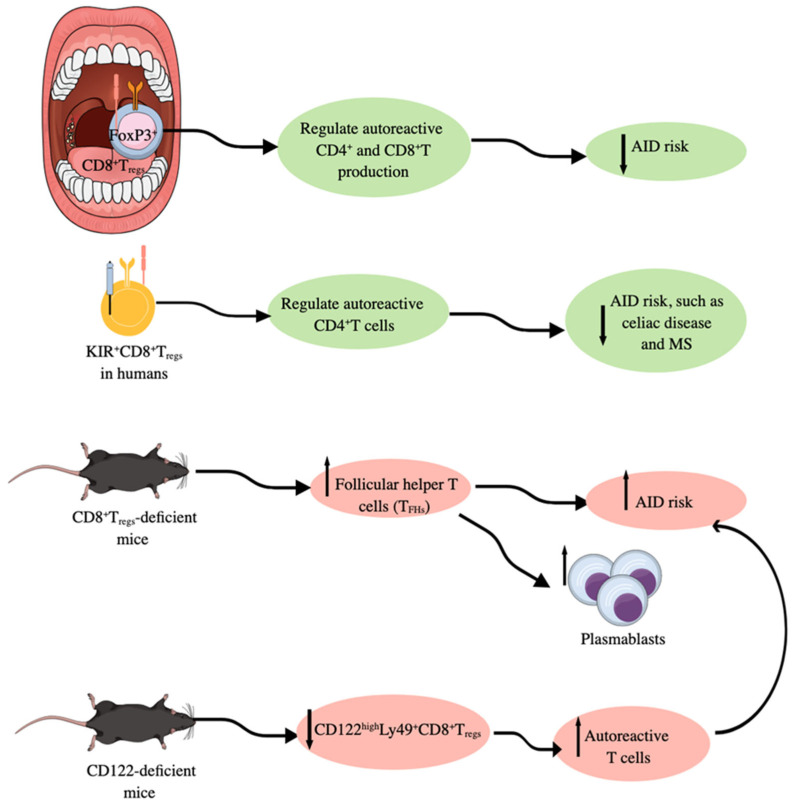
Foxp3^+^CD8^+^T_regs_ in immune homeostasis. In humans, Foxp3^+^CD8^+^T_regs_ present in SLOs, such as tonsils, regulate the production of autoreactive CD4^+^ and CD8^+^T cells; therefore, their deficiency is associated with an increased risk of AIDs. In addition to Foxp3^+^CD8^+^T_regs_, KIR^+^CD8^+^T_regs_ also regulate autoreactive CD4^+^T cells to lower the risk of AIDs. Moreover, Foxp3^+^CD8^+^T_regs_-deficient mice develop higher number of follicular helper T cells (T_FHs_) and develop AIDs, which is further supported by the increased number of plasmablasts responsible for autoantibody production. CD122-deficient/KO mice have lower numbers of CD122^high^Ly49^+^CD8^+^T_regs_ and develop higher numbers of autoreactive T cells responsible for AID development. Notable, mouse Ly49 corresponds to human KIR. Kindly see the text for details.

CD8^+^CTLs may also serve as regulatory T cells, like conventional CD25^+^CD4^+^forkhead box protein 3 (Foxp3)^+^ regulatory T cells (T_regs_), by exerting immunoregulatory functions [265,266]. Although CD25^+^CD4^+^Foxp3^+^T_regs_ and CD8^+^T_regs_ serve as immunoregulatory immune cells, they work differently. For example, in SLOs, such as human tonsils, CD25^+^CD4^+^Foxp3^+^T_regs_ negatively control autoantibody production upon stimulation with a panel of classical autoantigens (autoAgs) and also suppress high-affinity Ab production [267]. On the other hand, CD8^+^T_regs_ do not affect antibody production significantly but they do regulate the production of autoreactive CD4^+^ and CD8^+^T cells (Figure 4) [267]. For example, CD8^+^T_regs_-deficient mice exhibit an upregulated population of follicular helper T cells (T_FHs_) due to the escape of self-reactive T cells from the tolerance generation mechanism (Figure 4). However, GzmB KO mice with attenuated CD8^+^T_regs_ function have increased numbers of plasmablasts in response to upregulated T_FHs_ as an indirect effect (Figure 4) [267]. The different effects of CD25^+^CD4^+^Foxp3^+^T_regs_ and CD8^+^T_regs_ in immunoregulation are also sex-dependent; for example, tonsils from women with T_regs_ ablated exhibit the highest autoimmune response, which is also true for B cells obtained from unmanipulated tonsils [267]. Moreover, the high incidence of AIDs in women correlates well with different effects of CD25^+^CD4^+^Foxp3^+^T_regs_ and CD8^+^T_regs_ in immunoregulation in both sexes [268,269].

The CD8^+^Foxp3^+^T_regs_ are extremely rare in unmanipulated mice (0.07–0.4%) and healthy humans (0.1–1%) under homeostasis in their peripheral circulation [270,271]. CD8^+^Foxp3^+^T_regs_ are also present in the T cell zones surrounding germinal centers (GCs) of SLOs (LNs and tonsils) of healthy humans and express CCR7. The freshly isolated CD8^+^CD39^+^CD26^−^ cells act as effective suppressor cells, comparable to ex vivo-induced CD8^+^CCR7^+^ T_regs_ in humans, and suppress CD4^+^T cells [271]. Notably, young (<30 years old) and aged (>60 years old) humans have the same frequency of circulating CD8^+^CCR7^+^Foxp3^+^T_regs_ but older donors’ CD8^+^CCR7^+^T_regs_ have lost almost all of their CD4^+^T cell suppressor activity due to loss of NADPH oxidase 2 (NOX2), which is critical for reactive oxygen species (ROS) production [271]. This provides another reason for a dysregulated immune response in older adults, depending on the disease type, as the loss of NOX2^+^CD8^+^T_regs_ appears to be disease-specific but not solely due to the systemic inflammation seen in older adults more frequently than in the younger population. However, in patients with type 1 diabetes mellitus (T1DM, a metabolic disease with local (pancreas) systemic inflammation), circulating CD8^+^Foxp3^+^T_regs_ show a decrease, which further decreases the severity and duration of the disease, independent of the patient’s age [272]. Thus, aging does not increase the number of CD8^+^CCR7^+^Foxp3^+^T_regs_ but does affect their quality of action, which further decreases during inflammatory disease. Further studies are needed in this direction.

CD8^+^Foxp3^+^T_regs_ have been identified during graft versus host disease (GVHD) reactions and regulate allotransplant-associated immune responses by lowering its severity, but are less potent immunosuppressors than conventional CD25^+^CD4^+^Foxp3^+^T_regs_ [273,274]. In addition to GVHD, CD8^+^Foxp3^+^T_regs_ can be induced and expanded in other inflammatory conditions, such as cancers, AIDs, CVIs, and TCR transgenic adoptive transfer models in mice with therapeutic potential as mentioned in detail elsewhere [271,275,276,277]. CD8^+^Foxp3^+^T_regs_ lacking Bcl-2-interacting mediator of cell death (Bim, a proapoptotic protein) protein exert better immunoregulatory function than wild type (WT) CD8^+^Foxp3^+^ T_regs_ due to their prolonged survival [273].

In addition to CD8^+^Foxp3^+^T_regs_, other CD8^+^Foxp3^−^T cells, such as CD8^+^CD103^+^ cells, CD8^+^CD39^+^CD103^+^ cells, CD8^+^CD28^−^ cells, and CD8^+^CD122^+^CD49d^+^ cells expressing PD-1 and IL-10, behave as immunoregulatory/immunosuppressive T cells in different inflammatory conditions, such as aging, CVIs, cancers, GVHD, and AIDs [266,278,279,280,281,282]. The tumor-specific CD8^+^CD103^+^ induced T_regs_ (iT_regs_) generated in the TME in response to TGF-β overexpress CTLA-4 and IL-10. However, they have very low levels of IFN-γ, TNF-α, and GzmB and exert tumor-supportive immunosuppressive action [280,283,284]. In naïve mice, CD122^high^Ly49^+^CD8^+^CTLs lacking Foxp3 expression have been identified as T_regs_, which depend on Helios (encoded by *IKzf2*) and Eomesodermin (EOMES) TFs and are produced in response to TGF-β [266,285]. Helios-dependent STAT5 activation is critical for their function and prevention of terminal T cell differentiation, and its deficiency is associated with B cell-dependent autoimmunity [285,286]. CD122^high^Ly49^+^CD8^+^T_regs_ are critical for germinal center (GC) formation, and they are not restricted to the non-classic MHC-1b Qa-1 (HLA-E in humans) molecule, like CD8^+^Foxp3^+^T_regs_. However, they can be derived by the recognition of activated T cells via classical MHC-1 αβTCR signaling and become IL-10 secreting active CD8^+^CD122^+^T_regs_ [211,287,288,289]. Moreover, Qa-1-deficient mice are more prone to develop AIDs, such as experimental autoimmune encephalomyelitis (EAE), an experimental model for a human AID called multiple sclerosis, due to the deficiency of Qa-1-restricted CD8^+^Foxp3^+^T_regs_ [290,291]. The TCR-dependent recognition of Qa-1-peptide complexes on target CD4^+^T cells is a critical immunosuppressive action of CD8^+^Foxp3^+^T_regs_ [292]. Furthermore, the Qa-1–CD94/NKG2A interaction disruption induces robust CD8^+^Foxp3^+^T_regs_ and NK cell activity against autoreactive CD4^+^T cells, which completely abolishes EAE development [292,293].

The immune-activated CD122^high^Ly49^+^CD8^+^T_regs_ have similar gene expression signatures to Qa-1-restricted CD8^+^Foxp3^+^T_regs_ [287]. Moreover, CD122^high^Ly49^+^CD8^+^T_regs_ are critically needed to maintain T cell immune homeostasis as CD122 (IL-2 receptor b chain or IL-2Rb) deficient mice are characterized by having abnormally high levels of autoreactive T cells (Figure 4) [294]. CD122^high^Ly49^+^CD8^+^T_regs_ are more potent immunoregulatory cells than CD4^+^Foxp3^+^T cells to protect from autoimmunity and GVHD [295,296,297]. PD-1 expression on CD122^high^Ly49^+^CD8^+^T cells determines their role as T_regs_ or central memory T cells. For example, PD-1^+^CD122^high^Ly49^+^CD8^+^T cells via PD-1-CD28 interaction secrete IL-10 and exert immunosuppressive action, whereas Ag-specific PD-1^−^CD122^high^Ly49^+^CD8^+^T cells act as bona fide memory T cells [298,299]. Interestingly, in humans, CD8^+^CXCR3^+^ T cells exhibit similar functions as mice CD122^high^Ly49^+^CD8^+^T_regs_, such as the increased production of immunoregulatory IL-10 with suppressed IFN-γ production [295,300]. Moreover, killer immunoglobulin-like receptor (KIR)-positive CD8^+^T cells target self-reactive helper CD4^+^T cells to protect against AIDs, such as celiac disease and multiple sclerosis (MS) (Figure 4) [301]. Human KIR^+^CD8^+^T cells are functionally and phenotypically like mice Ly49^+^CD8^+^T_regs_, as KIRs are evolutionarily equivalent to murine Ly49 receptors [211,301]. The selective Ly49^+^CD8^+^T cell depletion in mice with viral infection does not impact their antiviral function but induces AIDs after viral infection [301]. Thus, KIR^+^CD8^+^T cells, called CD8^+^T_regs_ are critical negative regulators of AIDs occurrence after the infection due to a process called molecular mimicry that induces AIDs in people recovered from certain infections [301,302,303,304].

### 3.4. Granzyme K^+^CD8^+^ Cytotoxic T Lymphocytes/Cells (GzmK^+^CD8^+^CTLs)

**Figure 5:**
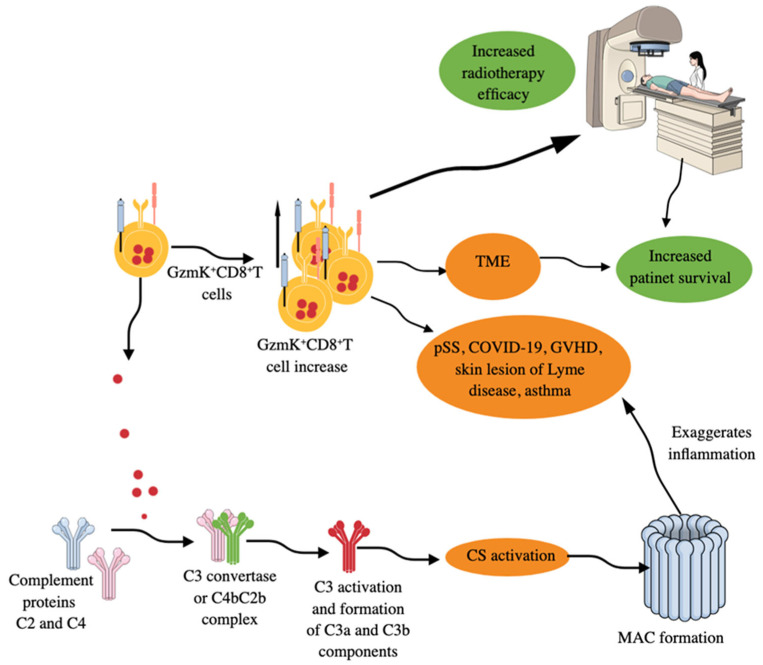
GzmK^+^CD8^+^T cells in inflammation and immune homeostasis. GzmK^+^CD8^+^T cells increase in number in patients with different AIDs, GVHD, allergic asthma, skin lesions of patients with Lyme disease, and TME of different cancers. Increased numbers of GzmK^+^CD8^+^T cells in TME correlates well with increased patient survival and increased efficacy of radiotherapy. Moreover, GzmK released from GzmK^+^CD8^+^T cells directly activates the complement system (CS) by cleaving C2 and C4 proteins of the CS. The cleaved C2 and C4 form a C4b–C2b complex (C3 convertase). C3 convertase cleaves C3 component into C3a and C3b and activates downstream complement cascades to form MAC. MAC formation further increases inflammatory processes, which can be lethal depending on the disease. Kindly see the text for details.

Granzyme K^+^CD8^+^T cells, which are low in GzmB and perforin, comprise the inflammatory CD8^+^T cells in inflamed tissues, such as synovium, synovial fluid, and several organs, such as gut, kidney, and bronchoalveolar lavage fluid (BALF) of COVID-19 patients (Figure 5) [305]. The size of the GZMK^+^CXCR6^+^CD8^+^T cell population also increases in the peripheral circulation of patients with primary Sjogren’s syndrome (pSS) (Figure 5), and their clones are shared with CD69^+^CD103^−^CD8^+^ tissue-resident memory cells (T_rms_) in the labial glands of these patients [306]. CD69^+^CD103^−^CD8^+^T_rms_ that highly express GZMK (GZMK^++^CD69^+^CD103^−^CD8^+^T_rms_) exhibit increased pro-inflammatory and cytotoxic properties in pSS compared to their CD103^+^ counterparts. The increased circulating GZMK^+^CXCR6^+^CD8^+^T cells in patients with pSS highly express CD122 and exhibit gene signatures like T_rms_ in these patients [306]. The constitutively elevated circulating IL-15 in patients with pSS polarizes circulating CD8^+^T cells into GZMK^+^CXCR6^+^CD8^+^T cells via a STAT-5-dependent signaling pathway. Thus, GzmK^+^CD8^+^CTLs also play a critical role in pSS immunopathogenesis via their pro-inflammatory and cytotoxic actions. Patients with bronchiolitis obliterans syndrome (BOS) during chronic GVHD (cGVHD) also show an increased population of circulating GZMK^+^CCR5^+^CD8^+^T cells (Figure 5), which are responsible for increased expression of fibrosis essential proteins, such as collagen type 1 alpha 1 chain (COL1A1) and fibronectin (FN1) in their fibroblasts [307]. These cells highly infiltrate the lungs of BOS-cGVHD mice and undergo clonal hyper-expansion there, with more cytotoxic, pro-inflammatory, migratory, and exhausted phenotypes. Bosutinib, a second-generation BCR–ABL1–tyrosine kinase inhibitor (TKI), has the potential to inhibit GZMK expression in CD8^+^T cells and its associated lung stiffness and pulmonary fibrosis in experimental cGVHD-BOS mice [307].

Moreover, skin lesions (erythema migrans, an early clinical manifestation of Lyme disease) of patients with Lyme disease, a tick-borne zoonosis caused by *Borrelia burgdorferi* (*B. burgdorferi*), have a clonally expanded population of GzmK^+^CD8^+^T cells secreting IFN-γ but exhibit less cytotoxic action (Figure 5) [308]. Thus, GzmK^+^CD8^+^T cells secreting IFN-γ also comprise one of the first immune cells to fight against the invading bacteria responsible for Lyme disease. These GzmK^+^CD8^+^T cells produce pro-inflammatory cytokines in response to both antigen-dependent and antigen-independent stimuli to exaggerate the inflammatory process but exert less cytotoxic action than conventional CD8^+^T cells [309]. However, GzmK may induce caspase-independent cell death by regulating different proteins, such as apurinic/apyrimidinic site endonuclease 1 (APEI), high-mobility group protein 2 (HMG2), and SET, components of the SET complex that sequesters the DNase NME1 [also known as NM23-H1, a GzmA-activated DNase (GAAD)] [310]. GzmK-mediated cleavage of SET components frees NME1 or NM23H1 to move to the nucleus, which induces single-stranded breaks in chromosomal DNA by abrogating the nucleosome assembly and degrading its inhibitor (IGAAD) SET to induce apoptotic cell death [310].

GzmK^+^CD8^+^T cells activate complement system (CS) by releasing GzmK (a tryptase like protease, which cleaves after arginine or lysine residues), which cleaves C2 and C4 CS proteins to form the central C3 convertase (C4bC2b complex), which cleaves C3 into C3a and C3b, resulting in the activation complement cascade and membrane attack complex (MAC) formation (Figure 5) [309,311,312,313]. These GzmK^+^CD8^+^T cells in the lungs further exacerbate the inflammatory symptoms of asthma via their GzmK-dependent proteolytic functions and CS activation (Figure 5), which was restored in animals treated with anti-GzmK drugs or not seen in GzmK KO mice subjected to asthma [314]. Moreover, GzmK, upon interaction with LPS, increases its binding potential to the CD14 and thus increases TLR4 signaling events to potentiate TLR-dependent pro-inflammatory signaling to aggravate the inflammation [315]. Interestingly, GzmK^+^CD8^+^T cells expressing CXCR4 (CD184) interact with CXCL-12 (stromal cell-derived factor-2 (SDF-1)) secreting fibroblasts to promote inflammation by stimulating the release of neutrophil chemoattractants from fibroblasts [316]. Thus, GzmK^+^CD8^+^T cells exert their pro-inflammatory function directly and through GzmK-mediated CS, TLR4 signaling, and CXCR4–CXCL12 interactions, thereby exaggerating inflammation and chronic inflammatory diseases, including AIDs, such as rheumatoid arthritis (RA), and can be targeted to overcome these conditions [315,316,317]. Moreover, in patients with acute ischemic stroke (AIS), the number of circulating GzmK^+^CD8^+^CD27^+^CCR7^+^ T cells increases, which correlates with the severity of clinical symptoms and is driven by the activation of the splenic sympathetic nervous system (SSNS) [318]. Stroke-induced activation of SSNS releases splenic norepinephrine (NE), which, via the ADRB2 receptor on GzmK^+^CD8^+^CD27^+^CCR7^+^T cells, promotes their mobilization. The ischemic brain injury induces CCL19 on endothelial cells, attracting GzmK^+^CD8^+^CD27^+^CCR7^+^T cells into brain parenchyma via their CCR7 receptor. The infiltration of GzmK^+^CD8^+^CD27^+^CCR7^+^T cells increases neuroinflammatory damage and neurological deficits, as indicated by the transient middle cerebral artery occlusion (tMCAO) mouse model study [318]. Aging is a well established non-modifiable ischemic stroke risk factor, and stroke patients are biologically older than their chronological age [319,320].

Furthermore, GzmK^+^CD8^+^T cells increase in the aging population as an indicator of healthy aging and enhance the inflammatory functions of non-immune cells [321]. A recent study has shown the protective function of GzmK^+^CD8^+^T cells against neurodegenerative disease (ND), such as Alzheimer’s disease (AD), by depositing GzmK on microglia and promoting the clearance of phosphorylated Tau (pTau) protein to prevent its spread in the central nervous system (CNS) and blood [322]. GzmK^+^CD8^+^T cells, by secreting GzmK, also inhibit osteoclastogenesis in postmenopausal osteoarthritis (OA) via activating the p38 mitogen-activated protein kinase (MAPK) signaling pathway [323]. Thus, GzmK^+^CD8^+^T cells are also critical for aging-associated inflammatory diseases, including NDs.

GzmK-mediated extracellular signal-regulated kinases (ERK/1/2) and p38 MAPK activation has also been reported in response to its direction interaction with protease activated receptor-1 (PAR-1), cleaving its N-terminal domain to exert its proinflammatory action on different immune cells, such as endothelial cells, fibroblasts, and macrophages, which release different proinflammatory cytokines and chemokines (IL-6, IL-8 and MCP-1) and promote the expression of different adhesion molecules, such intercellular adhesion molecule-1 (ICAM-1) [324,325]. PAR-1 has been shown to contribute to the innate immune response during viral infections by cooperating with toll-like receptor 3 (TLR3, which recognizes double-stranded (ds) RNA) to activate p38MAPK and IFN-β and CXCL10 expression) [326,327]. Thus, it would be interesting to investigate the effect of extracellular GzmK on PAR-1 and TLR3 cooperative action during viral infections. The inflammatory action of intra- and extracellular GzmK has been discussed in detail elsewhere [328,329].

The increased GzmK expression in the breast cancer tumor microenvironment (TME) corresponds to a high number of antitumor T cells, which is associated with increased overall survival (OS) and recurrence-free survival (RFS) of the patients and increased efficacy of cancer immunotherapy (Figure 5) [330]. GzmK^+^CD8^+^T cells are also highly present in the TME of glioblastoma (GBM) patients [331]. Moreover, in non-metastatic colorectal cancer (CRC), the interaction between CD15^+^ neutrophils and CD8^+^T cells promotes their skewing toward GzmK^+^CD8^+^T cells, which, by decreasing E-cadherin expression on the intestinal epithelial cells, promotes CRC progression [332]. However, the extracellular GzmK also promotes the release of soluble vascular endothelial growth factor receptor 1 (sVEGFR1) from endothelial cells independent of PAR1 activation to inhibit angiogenesis in CRC, indicating that targeting the GrK–sVEGFR1–angiogenesis axis may serve as a novel approach to target CRC [333]. On the other hand, CD66b^+^ neutrophils infiltrating the CRC TME interact with antitumor CD8^+^T cells to increase their survival, proliferation, and differentiation to increase their anticancer activity and the development of CD45RO^+^/CD62L^+^ central memory T cells [334]. Thus, it will be interesting to investigate further the neutrophil phenotype-dependent interaction with CD8^+^T cells and the generation of antitumor or tumor-supportive CD8^+^T cells, including GzmK^+^CD8^+^T cells. However, GzmB^+^GzmK^+^CD8^+^T cells have also been reported in inflamed tissues of patients with RA and CRC, and the function of these double Gzm-positive (GzmB^+^GzmK^+^) CD8^+^T cells remains to be explored [305,332]. A recent study has indicated that in people living with HIV-1 (PLWHs), GzmB^+^GzmK^+^CD8^+^T are highly reactive and proinflammatory in action to induce associated systemic inflammation [335]. Hence, further studies in several other cancers will establish GzmK’s role in tumor immunity, such as turning a cold tumor into a hot one or vice versa, and increasing the efficacy and responsiveness of immunotherapy among patients with cancer. For example, GzmK expression has also been detected in innate-like T cells, comprising a population of innate memory-like cells in the TME, and are rich in non-lymphoid tissues, such as liver and adipose tissues, but are low or absent in cord blood [336].

### 3.5. CD4^+^ Cytotoxic T Lymphocytes/Cells (CD4^+^CTLs)

Under certain conditions, such as viral infections, AIDs, and different cancers, upon activation, CD4^+^ helper T cells also act as killer T cells known as CD4^+^ CTLs [337,338,339,340]. These CD4^+^CTLs also play a critical role in IgG4-related disease (IgG4-RD), which has an unknown cause and is characterized by highly fibrotic lesions, with dense lymphoplasmacytic infiltrates containing a preponderance of IgG4-expressing plasma cells [341,342]. In IgG4-RD, clonally expended CD4^+^CTLs promote fibrosis and inflammation by secreting pro-fibrotic cytokines, such as IL-1β, TGF-β1, and IFN-γ, and simultaneously, they secrete GzmB and perforin to promote cell/tissue damage [341,342,343]. Severe acute respiratory syndrome (SARS)-CoV-2- or COVID-19-associated inflammation has the potential to imprint a durable CD4^+^T cell memory that remains enriched for transcripts related to their cytotoxic function and for interferon-stimulated genes (ISGs), which may be due to the altered chromatin accessibility landscape [344]. Additionally, the increased complement component C3a production in patients with severe COVID-19 generates CD16^+^T cells, which also exert potent cytotoxic action via an immune-complex-mediated mechanism, independent of TCR signaling-dependent granulation and cytotoxicity, which has not been seen in other diseases [345]. These CD16^+^CTLs maintain their cytotoxic action beyond acute COVID-19, and in these patients, they have promoted microvascular endothelial cell injury and chemokines for neutrophil and monocytes. Thus, COVID-19-associated inflammation also reprograms helper CD4^+^T cells into cytotoxic CD4^+^T cells, which affects their long-term immune function due to an alteration in their proliferation action.

These CD4^+^CTLsare characterized by the expression of CD29 (a marker for cytotoxic T cells), NK group 2 member D (NKG2D), GzmB, and perforin [157,339,346]. However, they differ in their cytotoxic action from conventional CD8^+^ cytotoxic T cells. For example, CD4^+^CTLs mainly kill cells by a Fas–FasL interaction-dependent manner, whereas CD8^+^CTLs utilize their granule exocytosis mechanisms, comprising perforin and GzB release, which is a minor function of CD4^+^CTLs [347]. Moreover, CD4^+^CTLs need antigenic stimulation to increase and maintain their cytotoxic molecules, such as their perforin level, whereas CD8^+^ conventional CTLs constitutively express these cytotoxic molecules in their granules post-thymic development [348].

The molecular mechanisms behind the conversion of helper CD4^+^T cells into CD4^+^ cytotoxic T cells are not precisely known. However, they are closely related to Th1 cells secreting IFN-γ and regulated by EOMES and/or T-bet TFs for their differentiation [349,350]. EOMES expression in CD4^+^CTLs not only supports GzmB and perforin expression but also increases FasL (CD178 or CD95L) expression to support their cytotoxic functions [351]. T-bet expression also supports FasL (by repressing CD40L (CD154) up-regulation) and perforin (PRF1) gene expression to program CD4^+^T cells into CD4^+^CTLs [352]. Moreover, the suppression of helper T cell master regulator T-helper-inducing POZ-Kruppel-like factor (ThPOK), which suppresses the cytolytic program in MHC-II-restricted CD4^+^T cells during the post-thymic termination of helper T cell programming, prevents Runx-dependent differentiation toward the CD8^+^ lineage, resulting in CD4^+^CTL generation [353,354]. ThPOK is necessary and sufficient for CD4 lineage or CD4^+^ helper T cell development [355,356]. Interestingly, naïve CD4^+^T cells can also behave as cytotoxic T cells through genetic suppression of ThPOK expression [349]. However, Ikaros family TF, IKZF3 or Aiolos suppresses CD4^+^CTL programming by suppressing IL-2 sensitivity and STAT5-associated CD4^+^CTL gene targets, such as Eomes, effector molecules, and IL2Ra [357,358]. On the other hand, Ikaros zinc finger transcription factor Eos (Ikzf4) is a positive regulator of CD4^+^CTLs during viral infections, such as influenza A virus (IAV) infection [358]. Eos is an antagonistic TF against Aiolos or IKZF3 and vice versa. For example, Aiolos suppresses Eos expression by inhibiting the STAT5-mediated Ikzf4 or Eos promoter activation [358]. Interestingly, compared to CD4^+^Th1 cells, the CD4^+^ cytotoxic T cells highly express three TFs, such as ThPOK, Runt-Related TF 3 (Runx3), and GATA-3 [359]. Furthermore, effector memory cells expressing CD45RA (T_EMRA_) are highly enriched for CD4^+^CTLs, unlike central memory (T_CM_) and effector memory (T_EM_) cells [360].

Supercentenarians (people who have reached 110 years of age) serve as a great model of healthy aging, with an increased number of CD4^+^CTLs in their peripheral circulation [361]. CD4^+^CTLs isolated from supercentenarians exhibit substantial heterogeneity in their cytotoxicity levels and share a transcriptome like that of CD8^+^CTLs by utilizing the CD8 lineage transcription program while retaining CD4 expression. The supercentenarians’ CD4^+^CTLs also secrete IFN-γ and TNF-α upon antigenic stimulation [361]. The increased number of circulating CD4^+^CTLs in supercentenarians indicates the development of unusual characteristics in the circulating lymphocytes to fight against infections and diseases to support their healthy longevity. Further studies are needed in this direction.

The CD4^+^CTLs recognize viral peptide–MHC complex to kill infected cells, working in the immune surveillance of APCs. They also recognize cells that generally do not express MHC-II, such as those infected with cancer, viruses, and/or bacteria (*Mycobacterium tuberculosis*) [346,349]. CD4^+^CTLs are critical to take care of viral [such as Epstein-Barr virus (EBV), Herpes Simplex virus (HSV), and cytomegalovirus (CMV)] infections, which evade their MHC-I recognition to escape classic CD8^+^CTLs [349,362,363,364]. The cytotoxic mechanism of action of virus-specific cytolytic CD4^+^T cells may vary between species. For example, HSV-specific CD4^+^CTLs exert cytolytic antiviral responses via Fas–FasL-dependent mechanisms in mice, but in humans, it is mediated explicitly by granule exocytosis [365]. Thus, these species-specific functional differences in CD4^+^CTLs during viral infections must be further explored in other pathologies, such as autoimmunity and cancers. For example, alloantigen-specific CD4^+^CTLs also depend on granule exocytosis for their cytolytic action in humans but not on Fas–FasL interactions [366]. However, in the absence of granular perforin, human CD4^+^CTLs exhibit relatively low cytotoxic function via a Fas–FasL signaling-dependent manner against Fas-sensitive cells, such as IFN-γ stimulated cells. In contrast, perforin is a central cytotoxic molecule during Ag-mediated cytotoxicity, such as in viral infections [367].

The presence of cytotoxic CD4^+^T cells having CD8-lineage transcription factor Runx3, expressing the class I-restricted T cell-associated molecule (CRTAM) within the intraepithelial lymphocyte (IELs) compartment, seems protective against IBD or autoimmune colitis [349,368]. CRTAM^+^CD4^+^T cells serve as the precursors of cytotoxic CD4^+^T cells, as their cultivation leads to the production of cytotoxic CD4^+^T cells [369]. Moreover, ectopic CRTAM expression in T cells induces IFN-γ production, EOMES induction, expression of genes (GzmB and perforin) responsible for cytotoxicity, and cytotoxic function. These CRTAM^+^T cells migrate from the thymus to the mucosal surface, such as the gastrointestinal tract (GT), lungs and reproductive tract, and to inflammatory sites to serve as CD4^+^CTLs to maintain normal organ homeostasis via their critical role in maintaining immune homeostasis through clearing dead cells and pathogens, and controlling inflammation [369,370].

The tumor-specific CD4^+^CTLs exert antitumor action that can be leveraged to increase the efficacy of other available cancer immunotherapies [346]. The anticancer activity of CD4^+^CTLs partly depends on the expression and activation of signaling lymphocytic activation molecule (SLAM) family member 7 (SLAMF7 or CD139) [371]. However, with the cancer progression and development of immunosuppressive TIME, the anticancer action of CD4^+^CTLs decreases, and their increased number is associated with prolonged survival of patients with cancer, including high-risk neuroblastoma patients [372]. The detailed functions of CD4^+^CTLs in CVIs and cancers have been discussed elsewhere [346,373,374]. CD4^+^CTLs may also be involved in AIDs, for example, in patients with systemic lupus erythematosus (SLE), NKG2D^+^CD4^+^CTLs expressing GzmB and perforin 1 (PRF1) kill immunoregulatory T_regs_ in a NKG2D–NKG2DL interaction-dependent (major pathway) and Fas–FasL-dependent manner that aggravates the pro-inflammatory phenotype of the disease [375].

Commensal bacteria in the gut, dietary factors, and TFs are critical factors for the generation of CD4CD8αα T cells in the IEL compartments [376,377]. For example, β-hexosaminidase, a conserved enzyme of the Bacteroidetes phylum of commensal bacteria, is involved in the differentiation of CD4^+^T cells to CD4CD8αα T cells, which exert anti-inflammatory action to protect against gut inflammation due to the expression of IL-10 and Lag3 [378]. CD4CD8αα CTLs lack (or have very low) ThPOK but highly express Runx3 TFs, which is controlled by another upstream TF called T-bet, which is highly expressed in these cells [353,376,379,380,381]. Moreover, T-bet is critical for IEL precursors for their development, differentiation, and expansion in the periphery in the presence of IL-15 [381]. Aryl hydrocarbon receptor (Ahr), a nuclear TF, can also regulate the maturation process of CD4CD8αα cytotoxic T cells by impacting ThPOK and Runx3. The gut bacteria *Lactobacillus reuteri* metabolize gut tryptophan into an indole derivative that activates Ahr, which, via regulating ThPOK and Runx3, supports TCRαβ^+^CD4^+^CD8αα^+^ CTLs in the IEL compartment [382,383]. Any alteration in gut bacteria critical for TCRαβ^+^CD4^+^CD8αα^+^ CTL generation and function has the potential to dysregulate gut and immune homeostasis by promoting local and systemic inflammation due to the breach in the gut epithelial barrier and local immune response.

CD4CD8αα T cells in the IEL compartments of small and large intestines also serve as CTLs as they produce GzmB and perforin for their cytotoxic action to remove injured epithelial cells and pathogens to maintain gut homeostasis [353,376]. Moreover, they also serve as immunosuppressive cytotoxic T cells in the IELs to take care of gut inflammation, such as colitis, to maintain immune homeostasis [376,381]. For example, the number of CD4CD8αα T cells in the IEL compartments of patients with inflammatory bowel disease (IBD) is significantly lower than that of control subjects, indicating their critical role in maintaining gut homeostasis and the local immune balance to suppress exaggerated inflammation [384]. Furthermore, the altered gut microbiota in patients with IBD activates their cytotoxic function, which supports their pro-inflammatory action as indicated by the release of TNF-α, IL-15, and IFN-γ, and the upregulation of CD107a expression. However, it suppresses their anti-inflammatory action and inhibits their immunoregulatory role to maintain immune homeostasis [353,376]. During bacterial sepsis, the increased release of extracellular cold-inducible RNA-binding protein (eCIRP, an alarmin) increases the cytotoxic action of CD4CD8αα T cells in the IEL compartments, as indicated by the increased expression of GzmB and perforin that induces gut epithelial cell death and damage to increase gut permeability and further spread of the infection and inflammation [385]. Vitamin D receptor knockout (KO) mice also exhibit impaired/low CD4CD8αα T cells in their IEL compartments due to reduced T cell homing in their gut IELs, as indicated by reduced chemokine receptor 9 (CCR9) expression, and exhibit an inflammatory phenotype, such as colitis and IBD, in response to normal commensal bacteria [386,387,388,389,390]. Thus, CD4CD8αα CTLs in the gut IELs are critical cytotoxic T cells for maintaining gut epithelial barrier integrity and local immune homeostasis, which depends on their balance of cytotoxic and anti-inflammatory functions.

## 4. CD8^+^CTL Maintain Immune Homeostasis via Direct Cell Interaction or Their Immune Mediators

### 4.1. Impact of CD8^+^CTLs on APCs During Antigen Presentation

The MHC-1-peptide complex on APCs (macrophages, DCs, and B cells) interacts with TCR and co-stimulatory molecules to induce the cytotoxic activity of CD8^+^CTLs as discussed in the earlier section. It is interesting to explore the impact of this interaction on APCs. It has been found that the antigen-dependent APC–CD8^+^T cell interaction induces the NOD-like receptor protein 3 (NLRP3) inflammasome activation in APCs to induce the maturation and production of IL-1b through perforin release [391]. The released IL-1β acts on other APCs to suppress the expression of MHC-II molecules via the suppression of class II transactivator (CIITA) gene expression [392]. CIITA serves as a master regulator of constitutive and IFN-γ-inducible MHC-II expression via two different mechanisms: serving as a transcriptional activator and as a general TF [393,394,395,396,397]. This MHC-II expression suppression will increase the MHC-I expression on APCs to support the function of CD8^+^CTLs. Reciprocally, increased release of IL-1b from APCs will increase their pro-inflammatory activity to take care of invading viral infections and cancers but may prove lethal during autoimmunity and GVHD. Moreover, effector and memory CD4^+^T cells abolish macrophage inflammasome-mediated caspase-1 activation and subsequent IL-1β release via direct interactions and indirectly via downregulating P2X7R-mediated NLRP3 inflammasome activation [398,399]. This further indicates that IL-1β is a negative regulator of MHC-II expression as effector and memory CD4^+^T cells abolish its release from APCs to support the validity of their immunoregulatory functions.

### 4.2. Immune Mediators Released from CD8^+^CTLs and Their Role in Mediating Immune Homeostasis

#### 4.2.1. IFN-γ

IFN-γ is a type II IFN released from activated/effector CD8^+^CTLs during an antiviral and anticancer immune response that also serves as a critical inflammatory signal for the expansion of CD8^+^CTLs along with IL-12 and type 1 IFNs (IFN-α and -β) [400]. However, IFN-γ-mediated stimulatory signals are required very early during the CD8^+^CTLs-mediated immune response, and they lose their responsiveness by downregulating the expression of IFN-γR2, which persists until the end of their expansion phase [401,402]. The avoidance of IFN-γ recognition by CD8^+^T cells during their later expansion phase protects them from its proapoptotic effect via the process called activation-induced cell death (AICD) [403,404,405]. IFN-γ induces apoptosis or AICD by inducing caspase 8 (CASP8) expression. IFN-γ induced apoptosis or AICD has also been observed in TME CD8^+^T cells, where IFN-γR2 expression negatively impacts their antitumor action and decreases the efficacy of ICIs in patients with metastatic melanoma. The decreased antitumor action is due to the inhibition of maintenance, clonal diversity, and proliferation of stem-like T cells, which are crucial for generating Ag-specific CD8^+^T cells [406,407,408]. Hence, loss of IFN-γ sensitivity at later stages is critical for successful antitumor immunity and ICI efficacy. Moreover, IFN-γ supports the expansion of low avidity CD8^+^T cells and reinforces high avidity CD8^+^T cells to enter the memory pool [405,409,410]. Thus, IFN-γ regulates CD8^+^ memory T cell differentiation and their survival even in the presence of weak TCR signals via IFN-γR signaling [410,411]. IFN-γ signaling in CD11b^+^ myeloid immune cells (MICs) is also sufficient to control CD8^+^T cell expansion and promote their contraction or AICD [412]. IL-12 and IL-18 secreted from MICs and APCs are critical IFN-γ-inducing cytokines [413,414,415]. Thus, at early stages of infection, IFN-γ suppresses CD11b^+^ MICs to limit their inhibitory action on the CD8^+^T cell expansion process.

Besides supporting the expansion of CD8^+^T cells, IFN-γ also supports the clonal expansion and survival of CD4^+^ helper T cells, which occurs independently of signal transducer and activator of transcription 1 (STAT1, a transcription factor that controls the expression of various IFN-γ-targeted genes) activation [416]. IFN-γ critically helps in the contraction and phenotype of Ag-specific CD4^+^T cells generated during infections [417]. Moreover, IFN-γ plays a significant role in the maintenance of effector memory CD4^+^T cells as they highly express T-bet TF, which is a critical target of IFN-γ [418,419,420]. Resting memory CD4^+^T cells express T-bet in their nuclei and IFN-γ further increases its expression, indicating initial polarization and subsequent imprinting of CD4^+^ Th1 cells. This process needs interlinked, sequentially acting positive feedback loops of TCR–IFN-γ–STAT1–T-bet and IL-12-STAT4-T-bet signaling events [419,421]. Thus, IFN-γ is a critical cytokine/IFN that supports CD8^+^T and CD4^+^T cell clonal expansion and the maintenance of their effector memory phenotypes to generate a rapid, robust immune response against the same antigen. However, chronic IFN-γ signaling compromises long-term survival of CD8^+^ memory T cells. It diverts self-renewing CD127^hi^ memory cell toward terminal differentiation to lower the number of CD8^+^ memory T cells, as is seen in MHC-II deficient mice, a phenomenon also observed in WT mice repeatedly exposed to IFN-γ [422]. This excess chronic IFN-γ is produced from endogenous colonic CD8^+^T cells in MHC-II deficient mice.

The mice deficient in IFN-γ production or defective IFN-γR expression are highly susceptible to recurring microbial (bacterial, parasitic, and viral) infections [423]. Similarly, human patients with mutations in their IFN-γR1 and IFN-γR2 genes are more susceptible to poorly virulent mycobacteria and bacille Calmette–Guérin vaccines, which become fatal to these patients in their childhood [423,424,425,426,427]. Moreover, IFN-γ-deficient children, along with an increased predisposition to mycobacterial infections and recurrent infections, exhibit defective neutrophil chemotaxis due to its direct effect on their different chemokine receptors, such as CCR1, CCR3, CCR6, and CXCR4; defective MHC-II expression; pro-inflammatory cytokines (TNF-α and IL-1β upregulation and IL-18 downregulation); reactive oxygen species (ROS) production; and decreased NK cell activity, indicating a critical role of IFN-γ in innate immunity and inflammatory process regulation [428]. For example, IFN-γ-mediated neutrophil regulation during tuberculosis infection is critical to control the pulmonary inflammation as it inhibits exaggerated pro-inflammatory neutrophil accumulation (neutrophilia) in *Mycobacterium tuberculosis* lung infection [429]. Moreover, systemic IFN-γ during AVIs, such as severe viral pneumonia, induces PD-L1 expression on bone marrow neutrophils to serve as regulatory neutrophils to provide a negative feedback loop to control the exaggerated inflammation [430]. Thus, IFN-γ not only acts as a pro-inflammatory IFN but also reprograms distant immune cells to control exaggerated inflammation during acute and chronic infections.

Along with affecting neutrophil functions and chemotaxis, IFN-γ increases the accumulation, activation, MHC-I expression, and cytotoxic action of NK cells not only during infections but also in cancers [431,432,433]. Hence, IFN-γ is critical for controlling microbial infections and TME to maintain immune homeostasis by controlling the expression of several target genes regulating the antimicrobial immune response, comprising innate and adaptive immunity [434,435,436,437]. Moreover, kidney-specific CD8^+^T cells-mediated release of IFN-γ in response to their activation due to salt retention via renal tubule cells has been shown to exacerbate hypertension [438,439]. Cell type-specific IFN-γ signaling has been discussed in detail elsewhere [440]. Hence, IFN-γ is critical for infectious and inflammatory diseases, including cancers and autoimmunity, and also for controlling non-inflammatory diseases, such as hypertension. However, patients with hypertension can have several upregulated inflammatory markers [441,442,443].

#### 4.2.2. IL-10

Fully differentiated effector CD8^+^T cells in their target organs also secrete a large amount of an anti-inflammatory cytokine called IL-10 at the site of infection or inflammation at the peak of their antiviral immune response to prevent exaggerated inflammation or tissue damage [444,445,446,447]. Type 1 IFNs are critical for the generation of IL-10 producing effector CD8^+^T cells during infections by promoting the production of IL-27 from local APCs, along with directly targeting effector CD8^+^T cells, which enhance the Blimp-1 (B-lymphocyte-induced maturation protein-1) and IRF4 TF expression required for IL-10 production [448]. The secreted IL-10 suppresses the pro-inflammatory actions of inflammatory innate immune cells, such as macrophages and neutrophils, and adaptive immune cells, including Th1 and Th17 cells [445,449]. Notably, IL-10 has immunostimulatory action on effector CD8^+^T cells by promoting the release of IFN-γ and their cytotoxic action [450]. However, there are IL-10 variants, as it is secreted as a non-covalent homodimer, which also suppresses effector CD8^+^T cell functions, but activates pro-inflammatory functions of macrophages [451,452]. Hence, the cell-specific pro- and anti-inflammatory functions of IL-10 depend on the IL-10 variant produced that binds to the two copies of IL-10 receptor heterodimer complex [two IL-10Rα (high affinity) subunits and two IL-10β (low affinity) subunits] [451,452,453]. Therefore, the IL-10 variant availability in the inflammatory microenvironment determines its cell-specific pro- and anti-inflammatory activity. These IL-10-producing effector CD8^+^T cells disappear in later stages as the infection is controlled, but IL-10 producing CD4^+^T cells (in SLOs) persist, indicating the involvement of different mechanisms for IL-10 production by these two different cell types (CD8^+^ and CD4^+^T cells) [87]. Notably, IL-10^+^CD8^+^T effector cells are a reversible and transient form of effector CD8^+^T cells, which develop to check the exaggerated inflammation and clear the infection at its later stages. Hence, IL-10^+^CD8^+^T effector cells are critical to maintain immune homeostasis, which regulates exaggerated inflammation and tissue damage without affecting viral clearance during later stages of an infection.

#### 4.2.3. Chemokines Released from CD8^+^CTLs and Their Role in Mediating Immune Homeostasis

The activated or effector CD8^+^CTLs release certain chemokines, such as XC motif chemokine ligand 1 (XCL1 or lymphotactin) and CCL5, or when regulated upon activation, normal T cells express and presumably secrete (RANTES) to recruit additional myeloid immune cells (MICs), like plasmacytoid DCs (pDCs) and macrophages, which further regulate the intensity of their (CD8^+^CTLs) activation signaling by cross-presentation and XCR1^+^DC maturation in SLOs, such as LNs [454]. The pDC accumulation in LNs (the site of Ag encounters for CD8^+^T cells) occurs via CCR5–CCL5 interactions [454]. These XCR1^+^DCs are critical for producing effector and memory CD8^+^T cells, which are essential for their antiviral action against current and future infection with similar viruses [455]. Moreover, modified murine XCL1 called mXCL1–V21C/A59C, containing a second disulfide bond to stabilize its chemokine structure, exerts much more potent chemotactic and calcium mobilization activities than the wild type XCL1 [456]. Therefore, mXCL1–V21C/A59C can be used as a vaccine adjuvant for better antiviral and antitumor vaccines by enhancing the cytotoxic action of CD8^+^CTLs and generating memory CD8^+^CTLs by different mechanisms [456,457,458].

For example, intratumoral XCLI injection enhances CD8^+^CTLs’ antitumor activity by recruiting CXCL9 (a ligand for CXCR3) and XCR1-expressing conventional type 1 DCs, which, by interacting with CTLs (CD8^+^T and NK cells) increase their antitumor action in melanoma [457]. Interestingly, genetic ablation of XCL1 in mice decreases the cytotoxic CD8^+^CTL response as indicated by their reduced proliferation and IFN-γ [459]. Of note, XCL1 only attracts CD8^+^DCs (present in the mouse and known for their superior Ag cross-presentation) and CD144^+^DCs (the only human DCs in circulation, which express XCR1 and correspond to murine CD8^+^DCs as superior Ag cross-presenting DCs) but not CD8^−^DCs and CD144^−^DCs, and other CD8^−^ and CD144^−^ immune cells, including B cells, T cells, NK cells, etc. [459,460]. The increase in CD8^+^ (mice) and CD144^+^DCs (humans) mobilization at the site of active CD8^+^CTLs and Ag cross-presentation in response to the XCR1–XCL1 interaction occurs due to increased Ca^2+^ mobilization [460]. This XCL1–XCR1 chemokine system is highly conserved between mice and humans, where specific DCs expressing XCR1 exhibit chemotaxis toward XCL1-expressing CD8^+^T and NK cells for Ag cross-presentation and increasing their cytotoxic potential [461]. Hence, XCL1 is a critical chemokine that regulates the cytotoxic potential of not only CD8^+^T cells, but also NK cells. The details of the XCR1–XCL1 interaction and its impact on the immune system to maintain immune homeostasis during health and disease are discussed elsewhere [462,463].

On the other hand, CCL5 released from CD8^+^T cells upon Ag encounter not only increases their immediate response to that Ag by promoting APCs infiltration but is also critical for memory CD8^+^CTLs because these memory CD8^+^CTLs frequently release CCL5 upon Ag encounter [464]. On the other hand, CD8^+^T cell exposure to IL-4 decreases their potential to release CCL5 by inhibiting CCL5 mRNA transcription via the STAT6-dependent signaling pathway, which inhibits their cytotoxic potential but may polarize them into Th2 cells called Tc2s secreting Th2 cytokines as discussed previously [464]. However, this IL-4-dependent inhibitory action of CD8^+^CTLs is reversible. Thus, CCL5 expression in CD8^+^CTLs is not only critical for their cytotoxic action but also for memory CD8^+^CTLs against viral infections, such as HIV-1 and cancers, and its deficiency decreases their cytotoxic potential and polarizes them toward Tc2s. For example, CCL5^+^ memory CD8^+^CTLs are critical to control viral load in HIV-1-infected people taking antiretroviral therapy (ART) [465]. Whereas, in AIDs, such as primary Sjogren’s syndrome (pSS), increased CCL5 release from CCR9^+^CD8^+^CTLs contributes to the disease severity and its immunopathology [466]. Thus, CD8^+^CTLs-derived CCL5 is critical for their cytotoxic and pro-inflammatory function to maintain immune homeostasis.

Additionally, CD8^+^CTLs release CCL3 (macrophage inflammatory protein-1α or MIP-α) and CCL4 (MIP-1β), which may further optimize local Ag presentation by increasing APC infiltration at the infection/inflammatory site. This process is also regulated by their swarming through homotypic chemokine signaling [454,467]. Moreover, CCL3 is critical for antiviral effector (cytotoxic) function of CD8^+^CTLs due to promoting their migration to the site of infection and inflammatory phenotype (CCR5 and CXCR3, and IFN-γ expression and cytolytic function) [468]. Thus, the release CCL3 not only promotes APC infiltration at the site of infection but also promotes the differentiation of CD8^+^CTLs into effector CD8^+^CTLs and their migration to the site of infection. However, not only do first-time Ag-encountering CD8^+^T cells release CCL3 and CCL4, but also memory CD8^+^T cells upon cognate Ag recognition from DCs release CCL3, CCL4, and XCL1 controlled by IRF4 expression/activity [469]. For example, memory CD8^+^T cells exhibit Ag-dependent arrest when it is presented by CCR2^+^Ly6C^+^monocytes and then they secrete IFN-γ and chemokines. This secreted IFN-γ strengthens the chemokines-induced antimicrobial action of CCR2^+^Ly6C^+^monocytes for quick removal of pathogens [469]. Moreover, CCL3 and CCL4 also increase the antitumor activity of CD8^+^CTLs by increasing their infiltration at the tumor site and cytotoxic action [467,470]. Hence, chemokines released by CD8^+^CTLs not only regulate their cytotoxic and pro-inflammatory actions but also affect other immune cells to maintain immune homeostasis.

#### 4.2.4. Perforin and Gzms in Immunoregulation (Non-Cytotoxic Effects)

This section will only discuss the non-cytotoxic action of perforin and Gzms in immune regulation. Mice express more Gzms (GzmA, B, C, D, E, F, G, K, M, and N) than humans (GzmA, B, K, H, and M), and only GzmA exists as a homodimer; the rest are monomers [471,472]. GzmA, B, C, and/or F are critical for cytotoxic action via different mechanisms discussed elsewhere [473,474,475,476,477]. However, GzmA and B are not critical for spontaneous and experimental tumor rejection by cytotoxic T and NK cells [478]. In addition to exerting cytotoxic action, GzmA induces pro-inflammatory action in MICs, such as macrophages, and stimulates IL-1β, IL-6, IL-8 (macrophage inflammatory protein-2 or MIP-2), and TNF-α production and release due to CASP1 activation, which are critical for microbial clearance and maintaining pro-inflammatory actions of other immune cells, including CD8^+^CTLs [479,480]. GzmA also supports the phagocytic action of macrophages. Moreover, GzmA also induces IL-6 and IL-8 production in fibroblasts and epithelial cells, helping them to take care of infections by stimulating their immunoregulatory functions as discussed elsewhere [481,482,483,484,485,486]. However, chronic GzmA exposure, as seen during chronic infections and inflammation, may lead to fibrosis due to an exaggerated inflammatory immune response. During acute infections, GzmA can aggravate the inflammatory tissue damage. Therefore, GzmA^−/−^ mice or mice treated with GzmA inhibitors escape from lethal inflammatory outcomes during acute bacterial infections, causing sepsis or lipopolysaccharide (LPS)-induced peritonitis, leading to systemic inflammation [480,487,488].

Cytotoxic T cells contribute as an important source of GzmA and GzmB during bacterial infections and sepsis, along with NK cells [489]. GzmB inhibition or knocking out GzmB reduces disease severity in autoimmune blistering disease as it mediates IL-8 secretion, lesional neutrophil infiltration, and neutrophil elastase activity [490]. Moreover, GzmB modulates CD4^+^T cell differentiation and function in the intestinal compartment to modify intestinal inflammation during gastrointestinal infections, such as *Citrobacter rodentium* in mice [491]. GzmB also participates in the inflammatory process seen during zoonotic parasitic infection caused by *Echinococcus granulosus* (sensu lato), causing cystic echinococcosis (CE) [492]. GrzmK also induces a pro-inflammatory immune response by stimulating the release of IL-1β, IL-6, and IL-8 by macrophages, fibroblasts, epithelial cells, and endothelial cells to take care of the infection by promoting immune cell infiltration and their pro-inflammatory actions [328]. The details of Gzms in human health and disease have been discussed elsewhere [139,140,328,493,494].

**Perforins** are cytolytic pore-forming proteins released by CD8^+^CTLs and NK cells [495]. In addition to direct cytotoxic action to clear infection and cancer/aberrant cells for maintaining immune homeostasis, perforins also exert some killing-independent immunoregulatory actions [495]. For example, during homeostasis or in the absence of any infection/disease, perforin and Fas knockout (KO) or perforin^−/−^Fas^−/−^ mice exhibit overaccumulation of highly active CD8^+^T cells in their liver and kidneys and die between the ages of five and twelve weeks [496]. Perforin is critical for AICD of CD8^+^T cells, and its deficiency increases the expansion of alloreactive CD8^+^T cells, which cause a similar degree of GVHD as normal T cells after injection into sublethally irradiated C.B-17 severe combined immunodeficiency (SCID) mice [497,498]. The details of perforin in GVHD are discussed elsewhere [499]. Moreover, Staphylococcal enterotoxin B (SEB) injection in perforin^−/−^ mice selectively induces prolonged persistence of SEB-reactive CD8^+^T cells. Persistent or chronic viral infections (PVIs or CVIs) induce perforin-dependent depletion or anergy of Ag-specific CD8^+^T cells [500,501]. For example, anergic Ag-specific CD8^+^T cells of perforin-deficient mice overexpress antiapoptotic molecules (Bcl-2 and Bcl-XL or BCL2L) [501]. The expansion of perforin-deficient CD8^+^T cells during LCMV infection is not impaired. However, these effector CD8^+^T cells exhibit impaired function and succumb to intracerebral LCMV infection a few days later than their age-matched WT siblings [502]. The delayed mortality of intracerebral LCMV infection in perforin-deficient mice results from delayed recruitment of pro-inflammatory immune cells in the central nervous system (CNS). The LCMV infection-induced massive expansion of CD8^+^T cells and aberrant cytokine production are responsible for high mortality in vaccinated perforin^−/−^ mice due to vaccination [503]. Furthermore, the vaccine-associated mortality among perforin^−/−^ mice upon LCMV infection is due to epitope-specific memory CD8^+^T cells, which dictate the magnitude of secondary CD8^+^ T-cell expansion and exhaustion and the inability to regulate the production of CD8^+^ T-cell-derived IFN-γ, but is not due to vaccine modalities [503,504].

Moreover, perforin-deficient mice exhibit increased immune-mediated inflammatory damage due to the accumulation of overactivated Ag-specific CD8^+^T cells during CVIs or PVIs due to increased antigenic stimulation independent of viral load, and they do not live more than one month post-infection [500,505,506]. Interestingly, perforin is also critical to regulate TCMI to prevent autoimmunity or AIDs, as mice deficient in perforin and Fas develop spontaneous lymphoproliferative disease as seen in Fas-deficient (lpr) mice [500]. Perforin-deficient Fas^+^ mice are more prone to develop autoimmunity as indicated by exaggerated hypergammaglobulinemia, autoantibody (autoAb) production, and immune deposit-related end-organ damage than their WT counterparts [507]. Furthermore, perforin-deficient lpr animals die earlier than perforin-intact lpr mice due to overaccumulation of CD3^+^CD4^−^CD8^−^αβ T cells along with unaltered hypergammaglobulinemia, autoantibody production, and immune complex renal disease. This further supports perforin-dependent CD8^+^T cell regulation, which is critical to regulate systemic autoimmunity.

Perforin-deficient mice and humans develop fatal hemophagocytic lymphohistiocytosis (HLH) after infection due to impaired clearance of rare APCs (DCs) by CD8^+^T cells, which leads to continuous T-cell activation well beyond initial priming in later animals [508]. Thus, perforin deficiency impairs the selective cytotoxic “pruning” of DC populations by CD8^+^T cells, leading to their over-expansion with aberrant immunological functions. Another study has indicated that perforin^−/−^DC-Fas^−/−^ mice lead to over accumulation of DCs with higher Ag-presentation capacity to CD8^+^T cells, causing uncontrolled CD8^+^T cell expansion and activation with over IFN-γ production responsible for lethal HLH [509]. Thus, perforin and Fas are critical regulators of DC homeostasis, which critically regulate generation, proliferation, and overactive CD8^+^T cells responsible for inflammation and autoimmunity. Moreover, perforin^−/−^ mice subjected to collagen-induced arthritis show increased autoreactive peripheral CD8^+^T cells and are more susceptible to experimental autoimmune uveitis (EAU) [495,510]. Perforin deficiency increases inflammatory immune cell (CD8^+^T cells) infiltration and joint damage in a mouse model of monosodium urate (MSU)-induced gout [511]. These perforin-deficient infiltrated CD8^+^T cells exhibit high inflammatory action and do not kill infiltrated pro-inflammatory M1 macrophages but promote TNF-α release.

In addition to controlling Ag-specific DCs and CD8^+^T cells, perforin also controls the proliferation of CD4^+^T cells upon their TCR stimulation as their hyperproliferation is seen in perforin^−/−^ mice [495]. These hyperproliferative CD4^+^T cells show increased cell division and IL-2 production independent of their development in the thymus, spleen, and LNs. Thus, perforin deficiency only increases hyperproliferation of Ag-specific CD4^+^T cells, like Ag-specific CD8^+^T cells. The TCR stimulation of CD4^+^T cells obtained from perforin^−/−^ mice increases their intracellular calcium (Ca^2+^) influx, which enhances the nuclear factor of activated T cells-1 (NFAT1) TF activity, thereby supporting Th cell activity [495]. Perforin^−/−^ mice are more prone to develop non-alcoholic fatty liver disease (NAFLD) with increasing age than WT mice due to increased IFN-γ production by increased CD4^+^T cells in their liver [512]. Moreover, perforin deficiency supports pro-inflammatory M1 macrophage polarization of infiltrating monocytes in the liver of mice on a high-fat diet (HFD). It induces pro-inflammatory cytokine production from infiltrating CD8^+^T cells to induce HFD-induced NAFLD or non-alcoholic steatohepatitis (NASH) [513]. These perforin-deficient CD8^+^T cells do not exhibit cytotoxic action against bone marrow-derived M1 monocytes and macrophages during NASH. Moreover, vascular adipose tissues (VATs) of HFD-fed perforin^−/−^ mice show overaccumulation of IFN-γ-producing CD4^+^ and CD8^+^T cells due to their increased proliferation and impaired AICD and M1-polarized macrophages due to a lack of cytotoxic action of the accumulated defective pro-inflammatory CD8^+^T cells, increasing insulin tolerance and predisposition to type 2 diabetes mellitus (T2DM) [511]. Hence, perforin is critical to regulate homeostatic proliferation and the function of DCs, CD8^+^ and CD4^+^T cells to avoid their overaccumulation and functional dysregulation, which is responsible for immunopathologic symptoms during infectious and inflammatory diseases, and AIDs.

In addition to controlling cellular immunity or its homeostasis, perforin is also critical for Ab production. For example, perforin-deficient mice generate higher Ab titers upon vaccination against influenza than WT mice, along with higher IFN-γ production [514]. This increase in Ab production correlates with the increased proliferation, expansion, and function (IL-2 production) of Ag-specific CD4^+^T cells [495]. Thus, perforins are critical for immune homeostasis via their direct cytotoxic action and immune regulatory action independent of killing. Perforins also exert anticancer action via direct cytotoxic action and by increasing the efficacy of ICIs via their immunoregulatory mechanisms discussed in detail elsewhere [515,516].

## 5. Future Perspectives and Conclusions

T cells, including cytotoxic T cells, are programmed to maintain immune homeostasis during their development in the thymus. For example, tolerance to self-proteins or Ags and removal of self-destructive T cells, including cytotoxic T cells, occurs in the thymus. Moreover, newborns and children under the age of one year who have undergone thymectomies suffer from frequent episodes of allergies and infections, which are not common in people with an intact thymus. These children with thymectomies might also lack or have an abnormal population of CD8αα^+^IELs, which are critical for maintaining the functions of other IELs and intestinal immune homeostasis, necessary for protection against IBD and other inflammatory intestinal diseases, including gastrointestinal cancers, such as colorectal cancer [46,517,518]. For example, CD8αα homodimers of IELs recognize thymus leukemia (TL, a non-classical MHC-1 molecule) Ag expressed on intestinal epithelial cells (IECs) to maintain their partially activated phenotype, allowing them to respond immediately to gastrointestinal antigenic insults [519]. TL deficiency has expedited the colitis severity in a genetic mouse model of IBD due to defective CD8αα^+^IEL function failing to maintain local intestinal immune homeostasis. Therefore, it is critical to observe the long-term effects of thymectomies on the lives of children who have undergone this surgical procedure.

Moreover, to maintain immune homeostasis, CD8^+^CTLs acquire different Th phenotypes and functions depending on the disease phenotypes and tissue environment, which lowers their cytotoxic activity. For example, asthma exacerbation and increased frequency of asthma have been reported in people infected with pulmonary viral infections, such as respiratory syncytial virus (RSV), human rhinovirus (HRV), metapneumovirus, parainfluenza, and coronavirus infections in their childhood, which may be due to polarization of cytotoxic CD8^+^CTLs into Tc2s secreting Th2 cytokines with low or negligible cytotoxic actions, to avoid exaggerated inflammatory tissue damage [520,521].

Pulmonary influenza infection and COVID-19 have been shown to break the dormancy of breast cancer cells disseminated to different organs, such as the lungs. It causes their proliferation within days and a massive expansion of carcinoma cells into metastatic lesions within two weeks in a murine disease model [522]. IL-6 plays a critical role in this process and disseminates breast cancer cells, impairing T cell activation. For example, sustaining CD4^+^T cells under the burden of pulmonary metastasis of breast cancer is suppressed after pulmonary influenza infection, which further suppresses CD8^+^T cells and their cytotoxic action [522]. Thus, dysregulated CD8^+^T cells and their cytotoxic action during pulmonary influenza or SARS-CoV-2 infection increases the risk of cancer metastasis and associated mortality in humans compared to non-infected cancer survivors. For example, SARS-CoV-2 infection impairs memory T cell generation by inducing their frequent contraction, which is IL-6-mediated [523]. IL-6 induces the STAT-3-dependent central transcriptional program in CD8^+^T cells, supporting a pro-fibro-inflammatory program: limited cytotoxicity, diminished expression of tissue-protective inhibitory receptors (PD-1, LAG-3, and TIGIT), and augmented mucosal imprinting (CD103) [523]. Hence, efficiently working CD8^+^CTLs are not only required to clear the infection but also to return to the immune homeostasis stage to fight future infections and cancers, as their dysregulation predisposes infected people to metastasis and cancer-associated death.

Moreover, cytotoxic CD8αβT cells also play a crucial role in food allergies due to their effector functions in response to the food allergen [524]. The food-specific peripheral Tregs (pT_regs_) are induced to support nutrient absorption and commensal microbiota in response to activation by newly identified APCs expressing RORγt, such as type 3 innate lymphoid cells (ILC3s) [524,525,526,527]. These pT_regs_ interact with conventional type 1 DCs (cDC1s) under homeostasis conditions to limit the food Ag-specific CD8αβT cells. However, infections and food allergens disturb this balance, causing the expansion of food Ag-specific CD8αβT cells, which acquire effector responses against mimicked food Ags [524]. Therefore, tolerance to food Ags is acquired by the interplay between APCs and T cells, such as T_regs_ and CD8^+^T cells, and during infection, this circuit allows the transient expansion of a protective effector response without compromising the overall strategy to keep the food tolerance mechanism intact to ensure safe food consumption. Hence, CD8^+^CTLs are not critical to protect against infections, cancers, and other inflammatory diseases, but are also essential for food tolerance to maintain immune homeostasis as food is a critical component of metabolism and immunometabolism, regulating immune cell functions during health (homeostasis) and disease.

Now we can conclude that CD8^+^T cell programming is critical to maintain immune homeostasis, and their disturbed immune programming may predispose to a new disease as seen in people already suffering from a chronic disease, such as CVIs, AIDs, and cancers. The CD8^+^T cell programming governing their effector, memory, and exhaustion during different diseases is governed by their metabolic programming, epigenetic factors, APCs, inflammatory molecules, and neuronal factors, such as neurotransmitters (NTs), as they express different NT receptors (NTRs), including dopamine receptors [163,165,528,529,530,531,532]. CD8^+^T cell metabolism governing their phenotype and function during homeostasis and different diseases, such as infections and cancers, has been discussed elsewhere [165,528,533]. Moreover, complement component C1q also regulates the CD8 memory T cell subset, the mitochondrial metabolism of the so-called memory precursor effector cells (MPECs) by binding to expressed C1q receptor (C1qR) upon Ag recognition to lower the autoimmune response, including the generation of autoAbs in patients with systemic lupus erythematosus (SLE) [534,535]. On the other hand, C1q deficiency enhances short-lived effector CD8^+^T cell activity to induce lethal immunopathology during CVI [534]. Hence, CD8^+^CTLs and their effector phenotypes with and without cytotoxic actions (secreting various cytokines, chemokines, and IFNs) are critically needed to maintain immune homeostasis and protect the host from invading pathogens and dead or transformed cancer cells. Any functional dysregulation of CTLs may lead to the development of AVIs, CVIs, AIDs, and cancers, including rare autosomal recessive CD8 deficiency disorder with recurrent sinopulmonary infections later in childhood.

## Data Availability

Not applicable.
